# PAI-1, *MTHFR*, and FVL Thrombophilic Polymorphisms as Risk Factors for Fetal Growth Restriction: A Case–Control Study from a Tertiary Care Center

**DOI:** 10.3390/biomedicines14102238

**Published:** 2026-10-03

**Authors:** Răzvan-Cosmin Pană, Nicoleta-Loredana Anghel-Savciu, Marina Dinu, Dan Ruican, Ana-Maria Petrescu, Alexandra Oltea Dan, Elena-Anca Târtea, Andrei Greșiță, Diana-Ruxandra Hădăreanu, Diana-Rodica Tudorașcu, Georgică Târtea

**Affiliations:** 1 Department of Gynecology, University of Medicine and Pharmacy of Craiova, 2 Petru Rares St., 200349 Craiova, Romania; razvan.pana@umfcv.ro (R.-C.P.); marina.dinu@umfcv.ro (M.D.); ana.petrescu@umfcv.ro (A.-M.P.); 2 Department of Gynecology, Emergency County Hospital of Craiova, 1 Tabaci Street, 200642 Craiova, Romania; lore.anghel@yahoo.com; 3 Department of Obstetrics, Gynecology and IVF, HitMed Medical Center, 200130 Craiova, Romania; ruican.dan@hotmail.com; 4 Experimental Research Centre for Normal and Pathological Aging, University of Medicine and Pharmacy of Craiova, 2 Petru Rares Street, 200349 Craiova, Romania; alexandra.dan@umfcv.ro (A.O.D.); andrei.gresita@umfcv.ro (A.G.); georgica.tartea@umfcv.ro (G.T.); 5 Department of Neurology, University of Medicine and Pharmacy of Craiova, 2 Petru Rares Street, 200349 Craiova, Romania; 6 Department of Cardiology, University of Medicine and Pharmacy of Craiova, 2 Petru Rares Street, 200349 Craiova, Romania; diana.hadareanu@umfcv.ro; 7 Department of Medical Semiology, University of Medicine and Pharmacy of Craiova, 2 Petru Rares Street, 200349 Craiova, Romania; diana.tudorascu@umfcv.ro

**Keywords:** fetal growth restriction, hereditary thrombophilia, PAI-1 4G/5G, *MTHFR* C677T, factor V Leiden, placental insufficiency, small for gestational age, hypofibrinolysis

## Abstract

**Background/Objectives**: Fetal growth restriction (FGR) is one of the main causes of perinatal morbidity and mortality, and in most cases the mechanism is placental dysfunction of vascular origin. Hereditary thrombophilias have been evaluated as factors that favor maternal vascular malperfusion, but the data in the literature remain contradictory. The objective of the study was to evaluate the association between the PAI-1 4G/5G, *MTHFR* C677T and factor V Leiden G1691A polymorphisms and fetal growth restriction, as well as to analyze the dose–effect relationship between the number of thrombophilic variants and the severity of restriction. **Methods**: This was a retrospective case–control study including 480 singleton pregnancies: 240 cases with fetal growth restriction and 240 pregnant women with eutrophic fetuses, followed up for a period of five years. Cases were defined biometrically as an estimated fetal weight below the 10th centile, without Doppler confirmation, and the phenotype therefore corresponds to small-for-gestational-age rather than to the full consensus definition of fetal growth restriction; a secondary analysis restricted to cases below the 5th centile, performed in response to review, is reported. Maternal characteristics, genotypes, perinatal outcomes, and cumulative pattern of thrombophilic variants were analyzed. **Results**: PAI-1 4G allele carriers were significantly more frequent in the growth restriction group compared to the control group (70.8% vs. 44.6%; OR 3.02; 95% CI 2.07–4.40; *p* < 0.001), as were *MTHFR* C677T allele carriers (57.1% vs. 39.2%; OR 2.07; 95% CI 1.44–2.97; *p* < 0.001) and factor V Leiden carriers (33.3% vs. 12.1%; OR 3.64; 95% CI 2.27–5.83; *p* < 0.001). Carrier prevalence increased progressively with severity of restriction for the PAI-1 4G allele and, most steeply, for factor V Leiden (from 12.1% in controls to 75.0% below the 3rd centile; trend test *p* < 0.001); for the *MTHFR* 677T allele the increase was confined in the two case strata above the 3rd centile, and the estimate in the smallest subgroup was uninformative. The risk of growth restriction increased with the number of coexisting thrombophilic variants: OR 2.12 (95% CI 1.24–3.64) for one variant, OR 5.60 (95% CI 3.21–9.78) for two, and OR 24.42 (95% CI 7.87–75.78) for all three, compared with the absence of any variant. In multivariable analysis, the three polymorphisms remained independent predictors, with the strongest adjusted association being that of factor V Leiden (adjusted OR 3.33; 95% CI 2.03–5.47). Carriage of each variant was associated with a lower birth-weight centile: −12.3 points for PAI-1 4G, −10.2 for factor V Leiden and −8.0 for *MTHFR* C677T (all *p* < 0.001). **Conclusions**: In this study, PAI-1 4G/5G, *MTHFR* C677T, and factor V Leiden polymorphisms were independently associated with fetal growth restriction. The results are compatible with a contribution of hypofibrinolysis and inherited thrombophilia to the placental insufficiency phenotype, most clearly in the more severe strata, but they are associative and require confirmation in prospective cohorts incorporating Doppler assessment and placental histopathology.

## 1. Introduction

Fetal growth restriction (FGR) is the failure of the fetus to reach its genetically determined growth potential, as a result of a pathological process, most commonly placental dysfunction [1]. The consensus definition obtained through the Delphi procedure combines biometric criteria with Doppler abnormalities, differentiating restriction with early onset (<32 weeks) from that with late onset (≥32 weeks) [2,3]. FGR affects approximately 5–10% of pregnancies and is the second leading cause of perinatal mortality, after prematurity [1,4]. It is associated with a 5–10-fold increased risk of intrauterine death, iatrogenic prematurity, low Apgar score, acidosis at birth, hypoglycemia, necrotizing enterocolitis, and intraventricular hemorrhage [1,5,6]. Growth restriction is also associated with neurodevelopmental disorders and an increased risk of hypertension, obesity, type 2 diabetes, and cardiovascular disease in adulthood [1,7].

The pathophysiological changes in placental growth restriction are represented by maternal vascular malperfusion and include villous infarctions, perivillous fibrin deposits, distal villous hypoplasia, and spiral artery lesions [8]. The initiating mechanism is reduced spiral artery remodeling by the extravillous trophoblast, with the persistence of a vascular bed with increased resistance, pulsatile perfusion, and intervillous oxidative stress [9]. This imbalance is biochemically translated by an increase in the sFlt-1/PlGF ratio, a marker with demonstrated diagnostic value in fetal growth restriction [10].

The placenta is an organ with intense fibrinolytic activity, and the balance between plasminogen activators and their inhibitors determines both trophoblast invasion and the maintenance of blood fluidity in the intervillous space [11,12]. Plasminogen activator inhibitor type 1 (PAI-1, encoded by the *SERPINE1* gene) is the main physiological inhibitor of tissue-type and urokinase-type plasminogen activators, and its plasma concentrations increase several-fold during pregnancy, mainly through placental synthesis of PAI-2 and PAI-1 [11,12]. The 4G/5G insertion/deletion polymorphism in the promoter of the *SERPINE1* gene encoding PAI-1 directly influences transcription: the 4G allele, which does not bind a transcriptional repressor, is associated with significantly higher plasma levels of PAI-1 than the 5G allele [13]. Increased placental expression of PAI-1 has been documented in pregnant women with preeclampsia and fetal growth restriction since the 1990s [14], and the 4G/4G genotype has been correlated with placental insufficiency and fetal hemodynamic centralization [15,16]. On the other hand, methylenetetrahydrofolate reductase (*MTHFR*) catalyzes the conversion of 5,10-methylenetetrahydrofolate to 5-methyltetrahydrofolate, the methyl donor required for the remethylation of homocysteine to methionine [17]. The thermolabile variant C677T causes a reduction in enzymatic activity of approximately 35% in heterozygotes and up to 70% in homozygotes, with a consequent increase in homocysteinemia, especially in conditions of suboptimal folate intake [17,18]. Hyperhomocysteinemia induces endothelial dysfunction, oxidative stress, and a prothrombotic state, mechanisms implicated in placental vasculopathy [19,20]. Finally, factor V Leiden (*F5* G1691A) is the most common hereditary thrombophilia in European populations. The Arg506Gln substitution eliminates one of the cleavage sites of activated protein C, leading to resistance to factor Va inactivation and a persistent procoagulant status [21,22]. Allele frequency varies considerably by geography, being estimated at 2–7% in Europe, with higher values in parts of south-eastern Europe [22]; the corresponding carrier prevalence, which is the quantity usually reported in clinical series, is approximately twice the allele frequency. Regional data from south-eastern Europe remain scarce, and the carrier frequency in the Romanian population has not been established in a population-representative sample, which is one reason why the frequency observed in our control group requires explicit interpretation (Section 4.7).

Despite the accumulated knowledge, the association between hereditary thrombophilias and placental complications remains one of the most controversial topics in maternal-fetal medicine. Meta-analyses have reported significant associations between factor V Leiden and fetal growth restriction [23], while meta-analyses of prospective cohort studies have not confirmed these results [24], and large prospective cohorts, such as the study by Said et al. or the NICHD Maternal-Fetal Medicine Units network, have not shown a clinically relevant association [25,26]. Randomized trials of low-molecular-weight heparin prophylaxis in pregnant women with hereditary thrombophilias—TIPPS, FRUIT, ALIFE—have not demonstrated a consistent benefit on placental complications [27,28,29]. Accordingly, current guidelines from the American College of Obstetricians and Gynecologists and the American Society of Hematology do not recommend routine testing for inherited thrombophilias in pregnant women with a history of fetal growth restriction [30,31], and isolated *MTHFR* testing is explicitly discouraged by the American College of Medical Genetics practice guidelines and recent positions of the thrombosis and hemostasis societies [32,33]. This persistent discrepancy warrants further studies in geographically defined populations, especially in regions where the allelic distribution differs from that of Western cohorts and where thrombophilia testing is widely practiced. Population-based data from Romania are limited, although a recent study from the west of the country reported a high prevalence of *MTHFR* and PAI-1 4G/5G polymorphisms in pregnant women investigated for thrombophilia [34]. Regarding growth restriction itself, a prospective study conducted in a tertiary prenatal diagnostic center in Romania showed that the prenatal detection rate strongly depends on the severity and that early-onset and late-onset forms are distinguished by ultrasound features [35].

Most published series analyse thrombophilic variants in isolation; the cumulative effect of coexisting variants has rarely been quantified in a defined population, and this is the principal contribution of the present work. We hypothesised that the three variants would show a graded association with the severity of growth restriction and a cumulative association when coexisting. In this study, we aimed to compare the frequency of PAI-1 4G/5G, *MTHFR* C677T, and factor V Leiden genotypes between pregnancies complicated with fetal growth restriction and pregnancies with normal fetal growth, as well as to analyze the dose–effect relationship between the number of thrombophilic variants and the severity of restriction. We started from the hypothesis that the three variants will show a graded association with the severity of growth restriction and a cumulative effect when they coexist.

## 2. Materials and Methods

### 2.1. Study Design and Population

We conducted an analytical, retrospective, case–control study, which included 480 singleton pregnancies followed and completed within the Department of Obstetrics and Gynecology of the Craiova County Emergency Clinical Hospital, Romania, during the period 2021–2025. The study was conducted in accordance with the principles of the Declaration of Helsinki and was approved by the Scientific Research Ethics Committee of the University of Medicine and Pharmacy of Craiova (no. 163 of 13 June 2024), an institution to which the hospital where the study was conducted is affiliated. All participants signed informed consent before sample collection for genetic testing performed in a clinical context, and the ethics committee subsequently approved the secondary use of these data for research purposes.

The study group included 240 pregnant women whose pregnancy was complicated by fetal growth restriction, and the control group included 240 pregnant women with fetuses with growth appropriate for gestational age, selected during the same period. Participants were identified among pregnancies delivered in the department between 2021 and 2025 for which genotyping of all three polymorphisms had already been performed during clinical care. Testing was requested individually, on clinical indication, and carried out in external private laboratories accredited by the Romanian Ministry of Health and recognized by the hospital in which the pregnancies were monitored; the reports were retrieved from the patients’ hospital records. Because these investigations were ordered and reported outside the hospital laboratory information system, the total number of pregnancies delivered in the study period that were, and were not, genotyped could not be reconstructed retrospectively, and we are therefore unable to present a STROBE/STREGA flow diagram with source-population denominators.

We describe the sampling frame instead. Cases were consecutive pregnancies with a birth-weight centile below the 10th, complete genotyping for the three polymorphisms and complete perinatal data, after application of the exclusion criteria in Section 2.2. Controls were pregnancies with a birth-weight centile between the 10th and the 90th and complete genotyping, delivered in the same period and selected consecutively. We regard the absence of source-population denominators as an important limitation and discuss its likely direction in Section 4.7.

Because the control group was drawn from pregnancies with fetal growth between the 10th and 90th centile, after exclusion of multiple pregnancies, structural and chromosomal anomalies, TORCH infection, acquired thrombophilia, chronic kidney disease and antenatal anticoagulant or antiplatelet treatment, it is by construction depleted of the principal determinants of preterm birth and does not represent the general obstetric population. Cases and controls were nevertheless ascertained through the same departmental coding system. The sampling frame is summarised in Supplementary Table S1.

### 2.2. Inclusion and Exclusion Criteria

We defined the following inclusion criteria: singleton pregnancy; certain gestational age, established by measuring the craniocaudal length in the first trimester; maternal age ≥ 18 years; complete genotyping for the three studied polymorphisms; complete perinatal data.

The exclusion criteria were: multiple pregnancies; major fetal structural anomalies or chromosomal anomalies diagnosed prenatally or postnatally; documented congenital infections from the TORCH group; antiphospholipid syndrome or other known acquired thrombophilias; antithrombin, protein C or protein S deficiency; prothrombin G20210A mutation; anticoagulant or antiplatelet treatment prior to inclusion; chronic kidney disease; uncertain gestational age data. Preeclampsia and gestational hypertension in the index pregnancy were not exclusion criteria, and pregnancies complicated by hypertensive disease were therefore eligible for both groups.

Major structural anomalies were excluded by detailed morphological examination performed in the first trimester and repeated in the second trimester. Evaluation of the fetal heart included, in addition to the four-chamber view, visualisation of the outflow tracts, in accordance with the ISUOG practice guidelines for the sonographic screening examination of the fetal heart [36]. This extended protocol is the standing protocol of our unit and has been described previously (a study from the same institution and a partly overlapping calendar period) [37]; that report is cited here as documentation of local practice and not as evidence of diagnostic performance. Exclusion of congenital heart diseases is important in a study of growth restriction, as they are themselves a recognized cause of restriction and could have affected the attribution of the phenotype to placental dysfunction.

### 2.3. Definition of Fetal Growth Restriction and Severity Stratification

**Figure 1:**
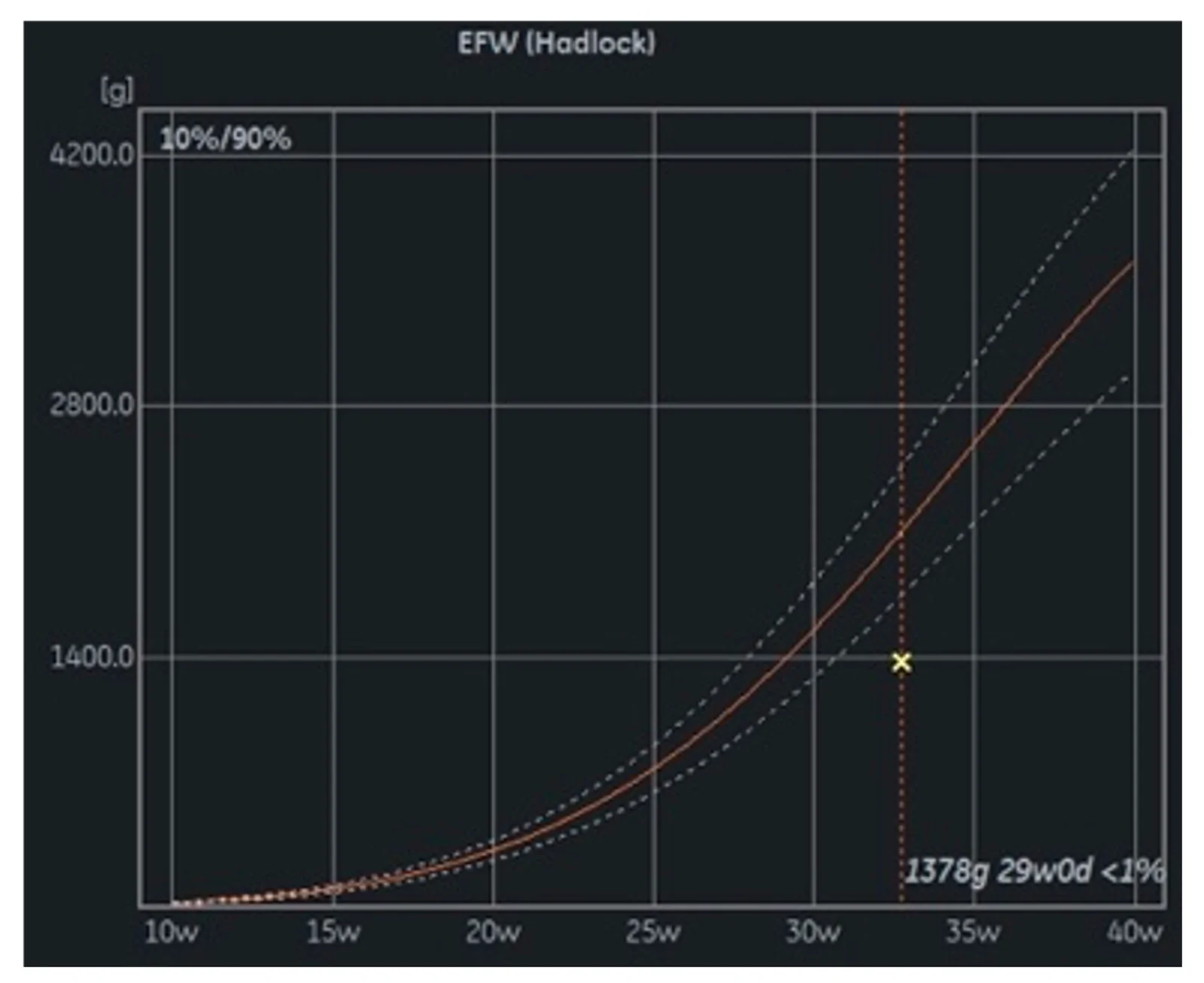
Fetal growth trajectory represented on centile curves generated by the ultrasound system (Hadlock method). Pregnancy complicated by severe fetal growth restriction, with estimated fetal weight below the 3rd centile.

Fetal growth was monitored by ultrasound according to the institutional protocol, with serial examinations at 2–4 week intervals depending on severity. Estimated fetal weight was calculated using the Hadlock method, implemented in the ultrasound software used (Voluson E10, GE Medical Systems Chicago, IL, USA), and centiles were referenced to the Hadlock fetal growth curves corresponding to gestational age [38,39] (Figure 1). The same reference was used to calculate the birth weight centile, on the basis of which the severity stratification was performed. The diagnosis of fetal growth restriction was established when the estimated fetal weight was below the 10th centile for gestational age [1]. We wish to be explicit about what this definition does and does not capture. The phenotype studied is small-for-gestational-age by biometric criteria; it does not incorporate the Doppler components of the Delphi consensus definition of fetal growth restriction, and it therefore includes an unknown proportion of constitutionally small fetuses, expected to be concentrated in the mildest stratum. Analyses restricted to cases below the 5th centile, in whom placental disease is more likely, are reported as a post hoc sensitivity analysis performed in response to review (Supplementary Table S2), and mechanistic interpretation is confined to those strata. The choice of reference curve substantially influences the proportion of fetuses classified as small for gestational age: international prescriptive standards, such as INTERGROWTH-21st [40], identify a population that is partly different from that identified by descriptive ultrasound standards, an aspect that is discussed in the Limitations Section.

Cases were stratified into three severity subgroups, depending on weight centile: 5th–10th centile (*n* = 151), 3rd–5th centile (*n* = 77), and below the 3rd centile (*n* = 12). The control group consisted of pregnancies with weight centiles between the 10th and 90th centiles.

Cases were additionally divided according to gestational age at delivery into those delivered before 32 weeks (*n* = 66) and at or after 32 weeks (*n* = 174). We emphasise that this is not equivalent to the early- versus late-onset classification of the FIGO document and the Delphi consensus, which is based on gestational age at diagnosis rather than at delivery, and that delivery before 32 weeks may itself result from clinical intervention; the subgroups are therefore designated throughout by gestational age at delivery and not as early- or late-onset growth restriction [1,2].

### 2.4. Genotyping

Genotyping was not performed in a research setting. All participants had been genotyped for the PAI-1 4G/5G (rs1799889), *MTHFR* C677T (rs1801133) and *F5* G1691A (rs6025) polymorphisms during clinical care, in five external private laboratories accredited by the Romanian Ministry of Health and recognised by the hospital in which the pregnancies were monitored. Throughout this article the rs1799889 insertion/deletion polymorphism is designated PAI-1 4G/5G, the designation used in the title, tables and figures; the gene in which it lies is *SERPINE1*. Gene symbols (*SERPINE1*, *MTHFR*, *F5*) are italicised; protein names (PAI-1) and the eponym factor V Leiden are not. Genomic DNA was extracted from peripheral venous blood and genotyping was performed by real-time polymerase chain reaction.

These laboratories issued genotype reports rather than run-level data. The investigators consequently had no access to extraction kits and catalogue numbers, instrument models, assay identifiers, reaction conditions, plate-level positive and no-template controls, first-pass call rates or DNA quantification and purity measurements, and we are unable to report them; this is stated as a limitation in Section 4.7. Because the tests had been requested for clinical indications before the present study was conceived, the laboratories were by construction unaware of the study and of the case–control status of the samples.

All genotype and perinatal records were independently re-checked against the original clinical charts by two investigators, and discrepancies were resolved by consulting the source laboratory reports; the final dataset contained no internal inconsistency between recorded carrier status and individual genotype.

For each polymorphism, both the complete genotype (homozygous normal, heterozygous, homozygous mutant) and the carrier status of the variant allele (heterozygous or homozygous mutant) were recorded.

### 2.5. Variables Analyzed

The following maternal variables were retrospectively extracted from the observation sheets: age at birth, parity, smoking status, pregestational body mass index, pregestational diabetes mellitus, chronic hypertension, autoimmune diseases, history of hypertensive disease in pregnancy, and gestational diabetes.

Perinatal variables included: gestational age at birth, mode of delivery (vaginal or cesarean section), birth weight, birth weight centile, 5 min Apgar score, need for neonatal respiratory support, and admission to neonatal intensive care for more than 24 h.

### 2.6. Statistical Analysis

Statistical analysis was performed with GraphPad Prism version 11.0.2 (GraphPad Software, Boston, MA, USA) for descriptive statistics, group comparisons, contingency tables, and trend tests, and with R version 4.4.1 (stats, car, and pROC packages) for multivariable logistic regression, multiple linear regression, ROC curves, and Hardy–Weinberg equilibrium testing. Normality of distribution of continuous variables was verified by the D’Agostino–Pearson test. Continuous variables are presented as mean ± standard deviation or median with interquartile range, and categorical variables as absolute frequencies and percentages.

Comparisons between groups were performed with Student’s t test for independent samples or the Mann–Whitney test, depending on the distribution, and for comparisons between the four subgroups with the ANOVA test with Tukey’s correction for multiple comparisons or the Kruskal–Wallis test with Dunn’s post hoc test. Categorical variables were compared by the χ^2^ test without continuity correction or, when at least one expected frequency was below 5, by Fisher’s exact test.

For each polymorphism, the odds ratio (OR) and 95% confidence interval were calculated by the Baptista–Pike method, both for the carrier (dominant) model and for the codominant, recessive and allelic models. The trend of genotype prevalence according to the severity of restriction was tested by the Cochran–Armitage test. Compliance with Hardy–Weinberg equilibrium was assessed in both the case and the control group, for all three polymorphisms, using both the χ^2^ test with one degree of freedom and the exact test of Wigginton et al.; results are reported in Supplementary Table S3. Independent predictors of fetal growth restriction were identified by multivariable logistic regression, including the three polymorphisms and maternal covariates, with the adjusted OR (aOR) and 95% confidence interval reported; the discriminative performance of the model was assessed by the area under the ROC curve. The determinants of weight centile and birth weight were analyzed by multiple linear regression, the latter with adjustment for gestational age at birth. Correlations were assessed by Spearman correlation coefficient.

Covariates for the multivariable model were chosen on clinical grounds and no stepwise or data-driven selection was used; the covariate set was not documented before the analysis. Multicollinearity was assessed by variance inflation factors, linearity in the logit for continuous covariates by the Box–Tidwell procedure, calibration by the Hosmer–Lemeshow test and the Brier score, and influential observations by Cook’s distance; pairwise interaction terms between the three polymorphisms were tested by likelihood ratio tests. The area under the ROC curve was internally validated by bootstrap resampling with 1000 replications and optimism correction. These analyses are reported in Supplementary Table S4 and Supplementary Figure S1. The single missing value in the dataset (chronic hypertension in one participant) was coded as absent; no imputation procedure was required.

Sample size and power. The study was assembled retrospectively and its size was determined by the number of eligible records rather than by a power calculation. For reference, detecting an odds ratio of 2.0 under the dominant model at a two-sided α of 0.05 with 80% power would require 133 participants per group for the PAI-1 4G allele (expected carrier prevalence in controls 45%), 133 for the *MTHFR* 677T allele (40%) and 245 for factor V Leiden (12%); the achieved sample of 240 per group meets that reference requirement for two of the three variants and falls marginally short for factor V Leiden, whose observed effect is considerably larger than the reference value. Post hoc power, computed from the carrier frequencies observed in the control group, was >99.9% for PAI-1 4G carriage (44.6%; OR 3.02), 97.8% for *MTHFR* 677T carriage (39.2%; OR 2.07) and >99.9% for factor V Leiden carriage (12.1%; OR 3.64), and remained 99.9%, 85.1% and 99.7% respectively after Bonferroni adjustment of α for the 14 tests. The study was underpowered for the recessive models: post hoc power was 13.6% for the *MTHFR* recessive comparison and 42.4% for TT versus CC. Details are given in Supplementary Table S5.

No analysis plan was registered before this study, which was assembled retrospectively from clinical records. The dominant model and the covariate set were chosen on clinical grounds before the analyses reported here were run, but were not documented in advance. The multiplicity corrections, power calculations, Hardy–Weinberg tests, model diagnostics, interaction tests, E-values and the analysis restricted to cases below the 5th centile were all performed in response to reviewers’ comments and are reported as exploratory. Supplementary Table S7 states, for every analysis in this article, when it was first performed and whether it was planned before the data were examined. Because several genetic models and multiple subgroup comparisons were examined, *p* values were adjusted for multiplicity by the Benjamini–Hochberg false discovery rate procedure, applied within each family of tests and, as a sensitivity analysis, across all 36 comparisons; Bonferroni-adjusted values are given in Supplementary Table S6. The dominant (carrier) model was chosen as the primary analysis for each polymorphism before the data were analyzed, and the remaining models are reported as secondary, exploratory analyses. Exact *p* values were computed by Fisher’s exact test for every comparison. The threshold for statistical significance was set at a two-sided *p* < 0.05 for unadjusted values and q < 0.05 for adjusted values.

## 3. Results

### 3.1. Clinical Characteristics of the Study Population

480 pregnancies were analyzed: 240 with fetal growth restriction and 240 controls. The distribution of cases by severity subgroups was as follows: 151 cases (62.9%) with centile 5–10, 77 cases (32.1%) with centile 3–5 and 12 cases (5.0%) below centile 3. The two groups were comparable for most maternal characteristics (Table 1). The mean maternal age was 26.40 ± 4.38 years in the growth restriction group and 26.22 ± 4.12 years in the control group (*p* = 0.550). There were no significant differences in parity (27.5% vs. 29.6%; *p* = 0.686), smoking (5.4% vs. 4.6%; *p* = 0.835), pregestational diabetes mellitus (5.4% vs. 5.4%; *p* = 1.000), chronic hypertension (2.1% vs. 2.1%; *p* = 1.000), or autoimmune diseases (0.8% vs. 1.7%; *p* = 0.686). Pregestational body mass index was significantly higher in the growth-restricted group (23.94 ± 2.65 vs. 23.26 ± 2.32 kg/m^2^; *p* = 0.002). A history of hypertensive disease in a previous pregnancy was almost three times more common in the growth-restricted group, without reaching statistical significance (5.8% vs. 2.1%; *p* = 0.058). Gestational diabetes was more common in the control group (5.0% vs. 1.2%; *p* = 0.032), an observation compatible with the growth-stimulating effect of maternal hyperglycemia.

### 3.2. Genotype Distribution and Association with Fetal Growth Restriction

**Figure 2:**
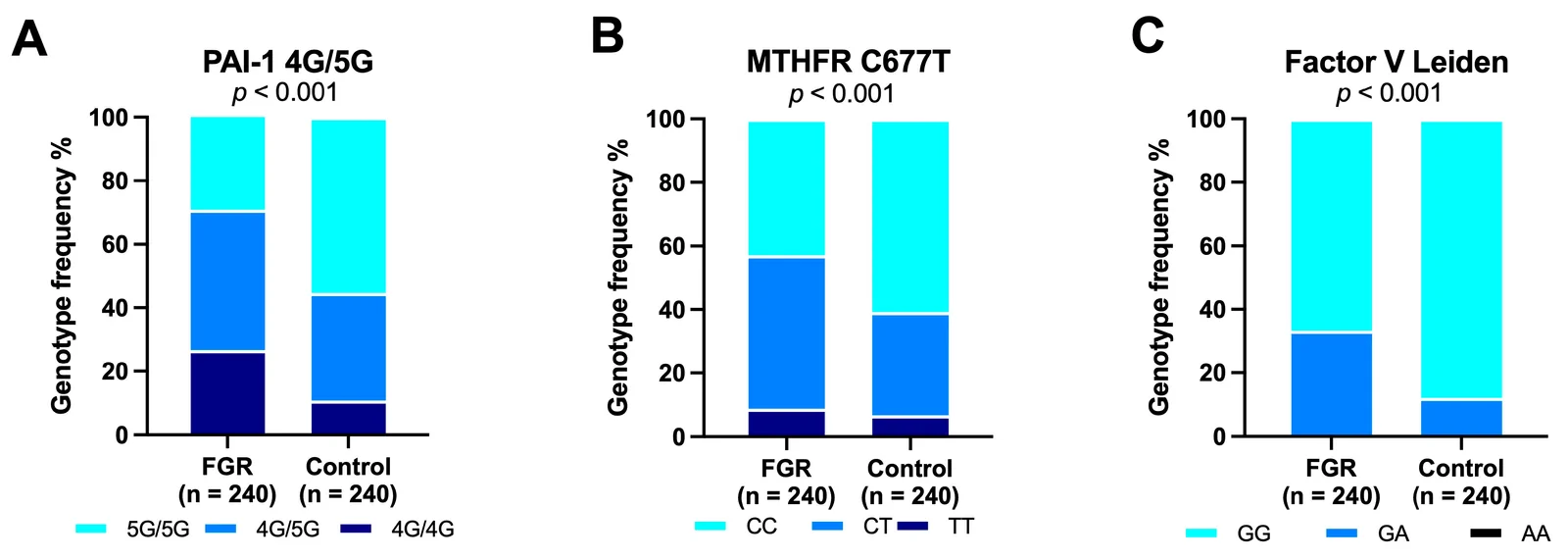
Genotype distribution of the PAI-1 4G/5G (**A**), *MTHFR* C677T (**B**) and Factor V Leiden G1691A (**C**) polymorphisms in the fetal growth restriction (FGR) group and in the control group (*n* = 240 per group). Bars show the percentage of each genotype within the group. In panel (**C**), the single homozygous AA case (0.4% of the FGR group) is not discernible at this scale. *p* values are from the χ^2^ test comparing the overall genotype distribution between groups.

**Figure 3:**
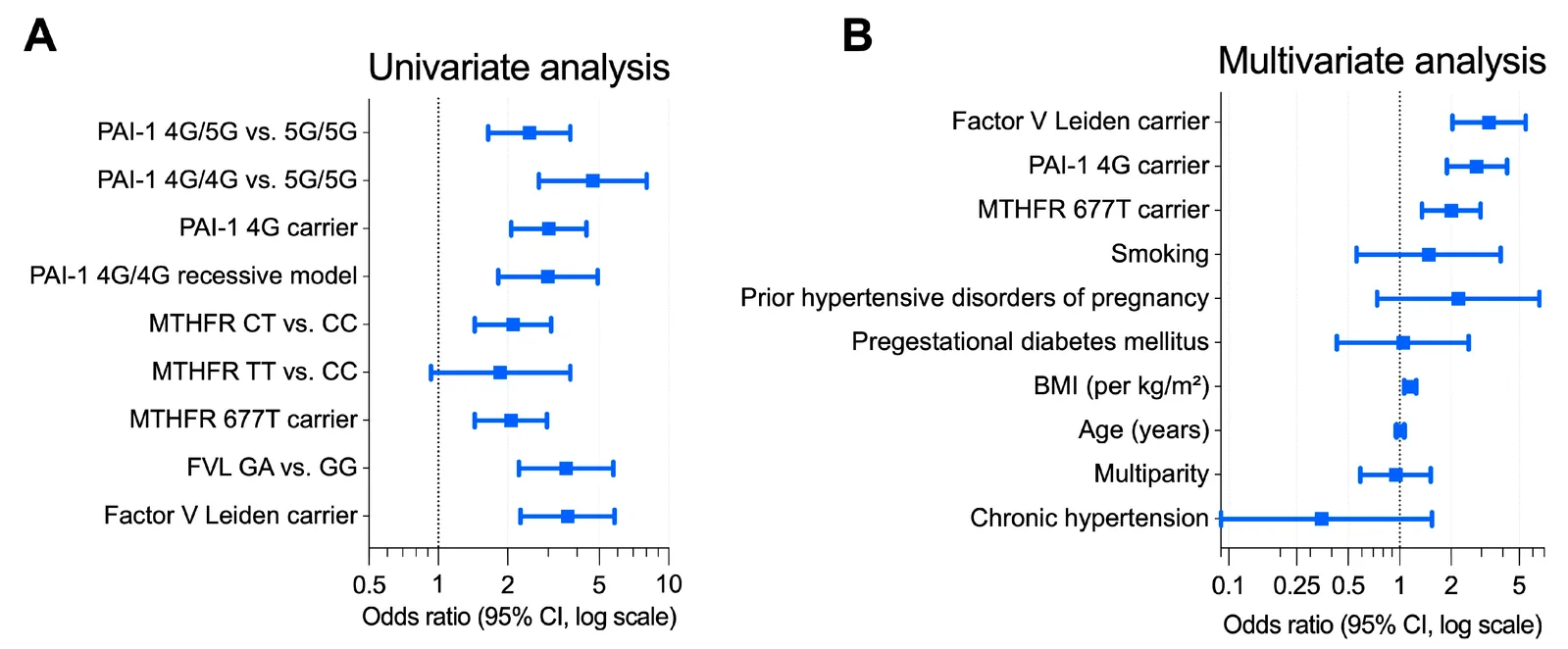
Forest plots of the association between thrombophilic polymorphisms and fetal growth restriction. (**A**) Unadjusted odds ratios from univariate analysis, shown for the codominant, dominant, recessive and allelic genetic models. (**B**) Odds ratios adjusted by multivariable logistic regression for maternal age, pre-gestational body mass index, smoking, parity, chronic hypertension, pre-gestational diabetes and prior hypertensive disorder of pregnancy. Squares represent point estimates and horizontal bars the 95% confidence intervals (Baptista–Pike method). The dotted vertical line marks the null value (OR = 1). The x axis is on a logarithmic scale. FVL, Factor V Leiden; BMI, body mass index.

The genotypic and allelic frequencies of the three polymorphisms are presented in Table 2 and Figure 2, and the summary of the odds ratios in Figure 3A.

PAI-1 4G/5G. The homozygous 4G/4G genotype was identified in 64 patients (26.7%) in the growth restriction group and in 26 (10.8%) in the control group, the heterozygous 4G/5G genotype in 106 (44.2%) and 81 (33.8%) patients, respectively, and the 5G/5G genotype in 70 (29.2%) and 133 (55.4%) patients, respectively. Carrier status of the 4G allele was significantly more frequent in the growth-restricted group (70.8% vs. 44.6%; OR 3.02; 95% CI 2.07–4.40; *p* < 0.001). In the codominant model, the risk was increased for both the heterozygous genotype (4G/5G vs. 5G/5G: OR 2.49; 95% CI 1.65–3.74; *p* < 0.001) and, more pronouncedly, for the homozygous genotype (4G/4G vs. 5G/5G: OR 4.68; 95% CI 2.73–8.03; *p* < 0.001). In the recessive model, the 4G/4G genotype remained associated with growth restriction (OR 2.99; 95% CI 1.82–4.92; *p* < 0.001). The frequency of the 4G allele was 48.8% in the growth restriction group and 27.7% in the control group (OR 2.48; 95% CI 1.90–3.25; *p* < 0.001).

*MTHFR* C677T. The CT genotype was present in 116 patients (48.3%) in the growth restriction group and in 78 (32.5%) in the control group, and the TT genotype in 21 (8.8%) and 16 (6.7%), respectively. Carrier status of the 677T allele was associated with growth restriction (57.1% vs. 39.2%; OR 2.07; 95% CI 1.44–2.97; *p* < 0.001). The association was mainly driven by the heterozygous genotype (CT vs. CC: OR 2.11; 95% CI 1.44–3.09; *p* < 0.001), while the homozygous TT genotype showed an effect in the same direction, but did not reach statistical significance (TT vs. CC: OR 1.86; 95% CI 0.93–3.74; *p* = 0.109), most likely due to the small number of TT carriers in both groups. In the recessive model, the TT genotype was not associated with growth restriction (OR 1.34; 95% CI 0.68–2.64; *p* = 0.494). The frequency of the 677T allele was 32.9% in the growth restriction group and 22.9% in the control group (OR 1.65; 95% CI 1.24–2.20; *p* < 0.001).

Factor V Leiden. The heterozygous genotype was identified in 79 patients (32.9%) in the growth restriction group and in 29 (12.1%) in the control group, and the homozygous genotype in one patient (0.4%) in the growth restriction group and in none in the control group. Carrier status was strongly associated with growth restriction (33.3% vs. 12.1%; OR 3.64; 95% CI 2.27–5.83; *p* < 0.001), the association also being confirmed for the heterozygous genotype compared to the normal genotype (GA vs. GG: OR 3.59; 95% CI 2.24–5.76; *p* < 0.001). The frequency of the variant allele was 16.9% in the growth restriction group and 6.0% in the control group (OR 3.16; 95% CI 2.02–4.93; *p* < 0.001).

In the control group, genotype distributions conformed to Hardy–Weinberg equilibrium for *MTHFR* C677T (χ^2^ = 1.54; exact *p* = 0.205) and *F5* G1691A (χ^2^ = 0.99; exact *p* = 1.000), with a significant departure only for PAI-1 4G/5G (χ^2^ = 5.96; exact *p* = 0.016), in the form of a deficit of heterozygotes (81 observed vs. 96.1 expected; fixation index 0.158). In the case group, distributions conformed for PAI-1 4G/5G (exact *p* = 0.072) and *MTHFR* C677T (exact *p* = 0.188), with a departure for *F5* G1691A (exact *p* = 0.005) consisting of an excess of heterozygotes, which is expected under the alternative hypothesis of a genuine association since cases are sampled conditionally on genotype. Full statistics are given in Supplementary Table S3. Four of the 36 comparisons lose significance under the global Bonferroni threshold while retaining it under the Benjamini–Hochberg procedure: carriage of PAI-1 4G/4G, of the *MTHFR* 677T allele and of factor V Leiden in the mildest severity stratum, and the comparison of one variant against none. All three primary associations survive both corrections (Supplementary Table S7d).

### 3.3. Association with Severity of Growth Restriction

**Figure 4:**
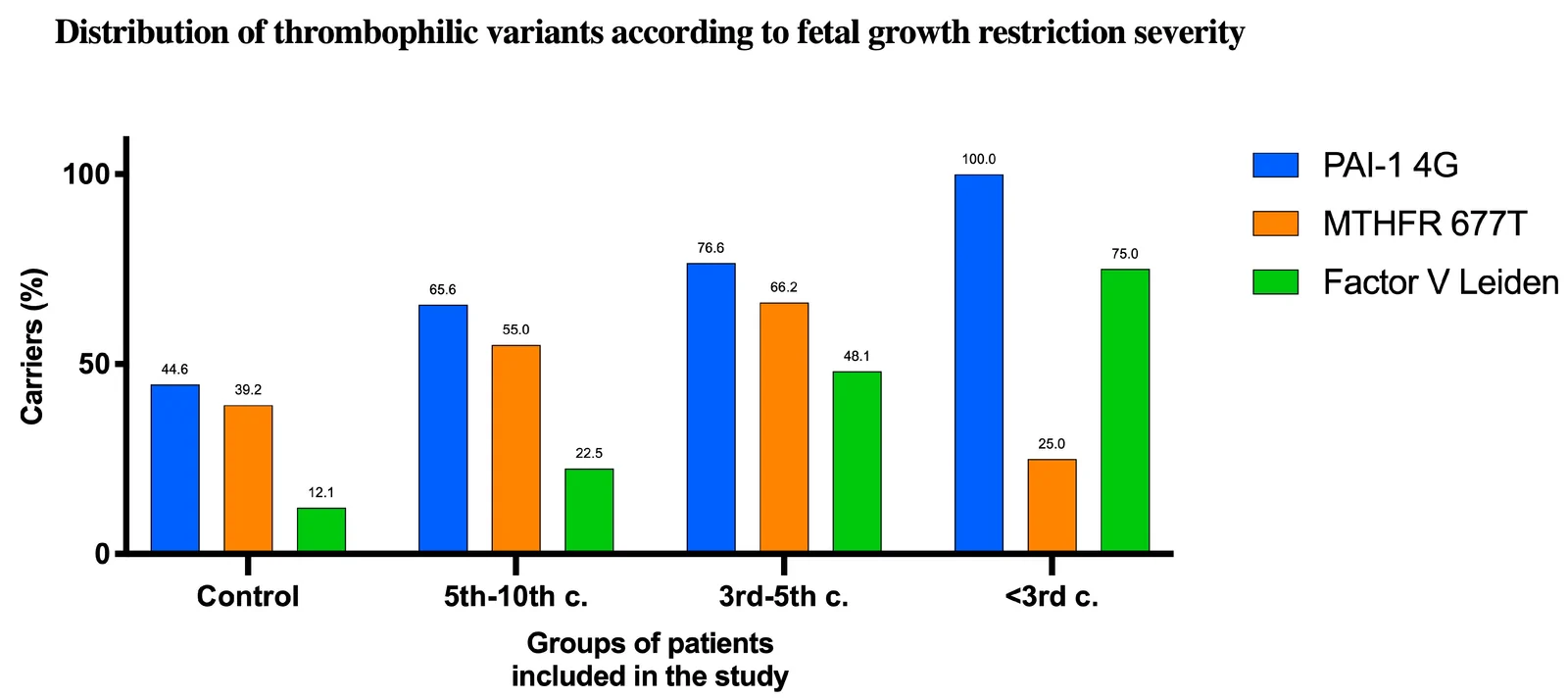
Prevalence of thrombophilic variant carriers across control subjects and the three subgroups of increasing fetal growth restriction severity (control, *n* = 240; 5th–10th centile, *n* = 151; 3rd–5th centile, *n* = 77; below the 3rd centile, *n* = 12). Cochran–Armitage test for trend across the four ordered categories: *p* < 0.001 for all three variants. Compared with controls (Fisher’s exact test): PAI-1 4G, *p* < 0.001 in all three subgroups; *MTHFR* 677T, *p* = 0.003, *p* < 0.001 and *p* = 0.380, respectively; Factor V Leiden, *p* = 0.007, *p* < 0.001 and *p* < 0.001, respectively.

A stratified analysis by severity subgroups is presented in Table 3 and Figure 4.

The prevalence of PAI-1 4G allele carriers progressively increased from 44.6% in the control group to 65.6% in the 5–10th centile subgroup (OR 2.37; 95% CI 1.55–3.61; *p* < 0.001), to 76.6% in the 3–5th centile subgroup (OR 4.07; 95% CI 2.27–7.32; *p* < 0.001) and to 100% in the subgroup below the 3rd centile (*p* < 0.001), with a highly significant Cochran–Armitage trend test (*p* < 0.001). A similar gradient was observed for the homozygous 4G/4G genotype, whose prevalence increased from 10.8% in the control group to 23.2% and 33.8% in the first two subgroups of cases (trend test *p* < 0.001); the estimate from the subgroup below the 3rd centile (25.0%; 3 of 12) is imprecise due to its small size.

Factor V Leiden showed the steepest gradient between severity strata: 12.1% in the control group, 22.5% in the 5th–10th centile subgroup (OR 2.11; 95% CI 1.23–3.64; *p* = 0.007), 48.1% in the 3rd–5th centile subgroup (OR 6.73; 95% CI 3.72–12.17; *p* < 0.001), and 75.0% in the subgroup below the 3rd centile (OR 21.83; 95% CI 5.58–85.31; *p* < 0.001), with a trend test of *p* < 0.001. In contrast to the other two variants, the magnitude of the association with factor V Leiden approximately doubled with each successive increase in severity.

For *MTHFR* C677T, the carrier prevalence was 55.0% in the 5–10th centile subgroup (OR 1.90; 95% CI 1.26–2.86; *p* = 0.003) and 66.2% in the 3–5th centile subgroup (OR 3.05; 95% CI 1.78–5.22; *p* < 0.001), with a significant trend in the two case strata above the 3rd centile (*p* < 0.001). In the subgroup below the 3rd centile, only 3 of 12 patients (25.0%) were carriers of the 677T allele, an estimate that did not differ significantly from the control group (*p* = 0.380) and should be interpreted as sampling variation, not as a protective effect. The homozygous TT genotype showed no trend between severity strata (trend test *p* = 0.634).

Comparison of pregnancies delivered before 32 weeks (*n* = 66) with those delivered at or after 32 weeks (*n* = 174) revealed no significant differences in the distribution of any of the polymorphisms: PAI-1 4G 72.7% vs. 70.1% (*p* = 0.752), PAI-1 4G/4G 31.8% vs. 24.7% (*p* = 0.327), *MTHFR* 677T 50.0% vs. 59.8% (*p* = 0.190), and factor V Leiden 28.8% vs. 35.1% (*p* = 0.443).

In the 3rd–5th centile stratum the point estimate for the homozygous 677TT genotype is inverse (OR 0.18; 95% CI 0.02–1.41; Supplementary Table S7b), in contrast to the positive association with carriage of the 677T allele in the same stratum. The contrast rests on a single case, does not reach the conventional threshold and shows no trend across strata; no inference is drawn from it.

### 3.4. Cumulative Effect of Thrombophilic Variants

**Figure 5:**
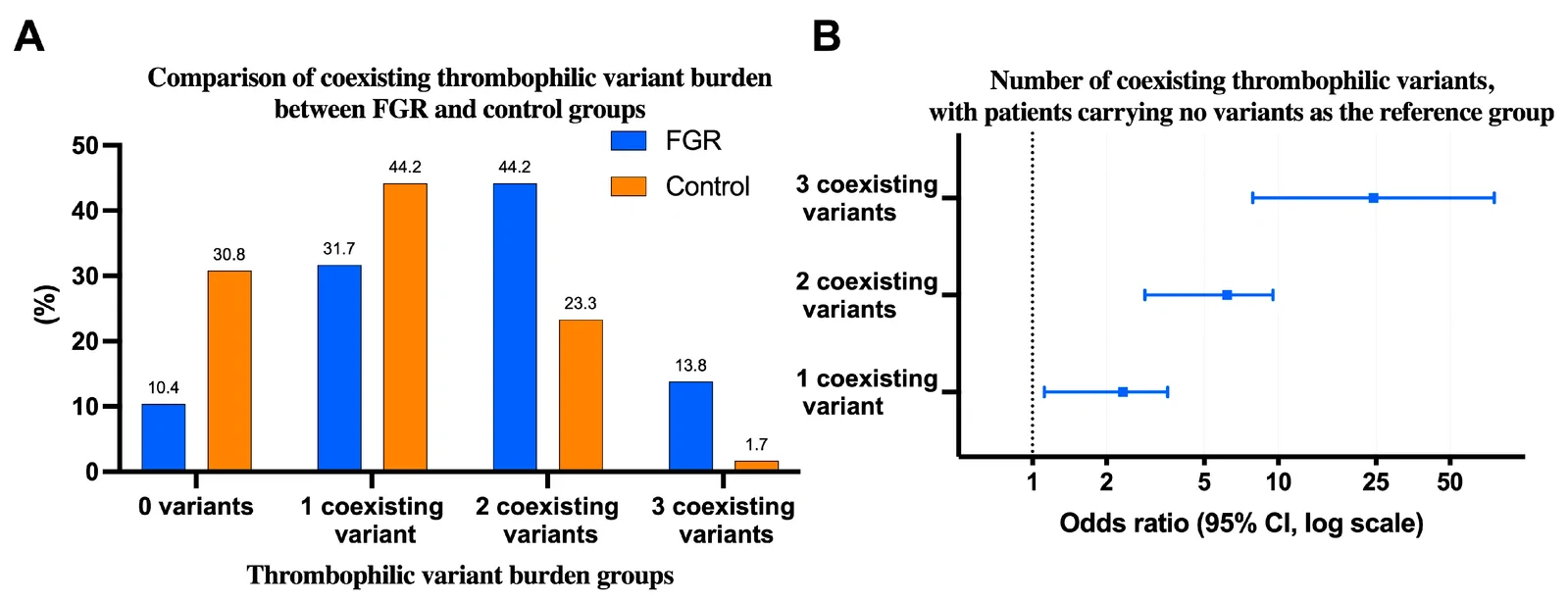
Cumulative effect of coexisting thrombophilic variants. (**A**) Distribution of the number of coexisting variants in the fetal growth restriction (FGR) and control groups (χ^2^ = 67.36; df = 3; *p* < 0.001). (**B**) Odds ratios for fetal growth restriction according to the number of coexisting variants, with women carrying no variant as the reference group: 2.12 (95% CI 1.24–3.64) for one variant, 5.60 (95% CI 3.21–9.78) for two and 24.42 (95% CI 7.87–75.78) for all three. Squares represent point estimates and horizontal bars the 95% confidence intervals; the dotted vertical line marks OR = 1. The x axis is on a logarithmic scale. The estimate for three coexisting variants is imprecise because only four control subjects carried all three.

The number of coexisting thrombophilic variants was associated with the risk of fetal growth restriction in a dose-dependent manner (Table 4, Figure 5). Compared with patients without any variant, the odds of growth restriction were 2.12 times higher in carriers of a single variant (95% CI 1.24–3.64; *p* = 0.006), 5.60 times higher in carriers of two variants (95% CI 3.21–9.78; *p* < 0.001), and 24.42 times higher in carriers of all three variants (95% CI 7.87–75.78; *p* < 0.001; overall χ^2^ = 67.36; df = 3; *p* < 0.001). The absence of any thrombophilic variant was three times more common in the control group than in the growth restriction group (30.8% vs. 10.4%).

Specific combinations of polymorphisms were also associated with growth restriction: PAI-1 4G together with *MTHFR* 677T (40.8% vs. 16.2%; OR 3.56; 95% CI 2.32–5.46; *p* < 0.001), PAI-1 4G together with factor V Leiden (27.1% vs. 5.4%; OR 6.49; 95% CI 3.46–12.14; *p* < 0.001), and *MTHFR* 677T together with factor V Leiden (17.5% vs. 6.7%; OR 2.97; 95% CI 1.62–5.45; *p* < 0.001). The concomitant presence of all three variants was identified in 13.8% of cases and 1.7% of controls (OR 9.41; 95% CI 3.28–27.00; *p* < 0.001).

**Figure 6:**
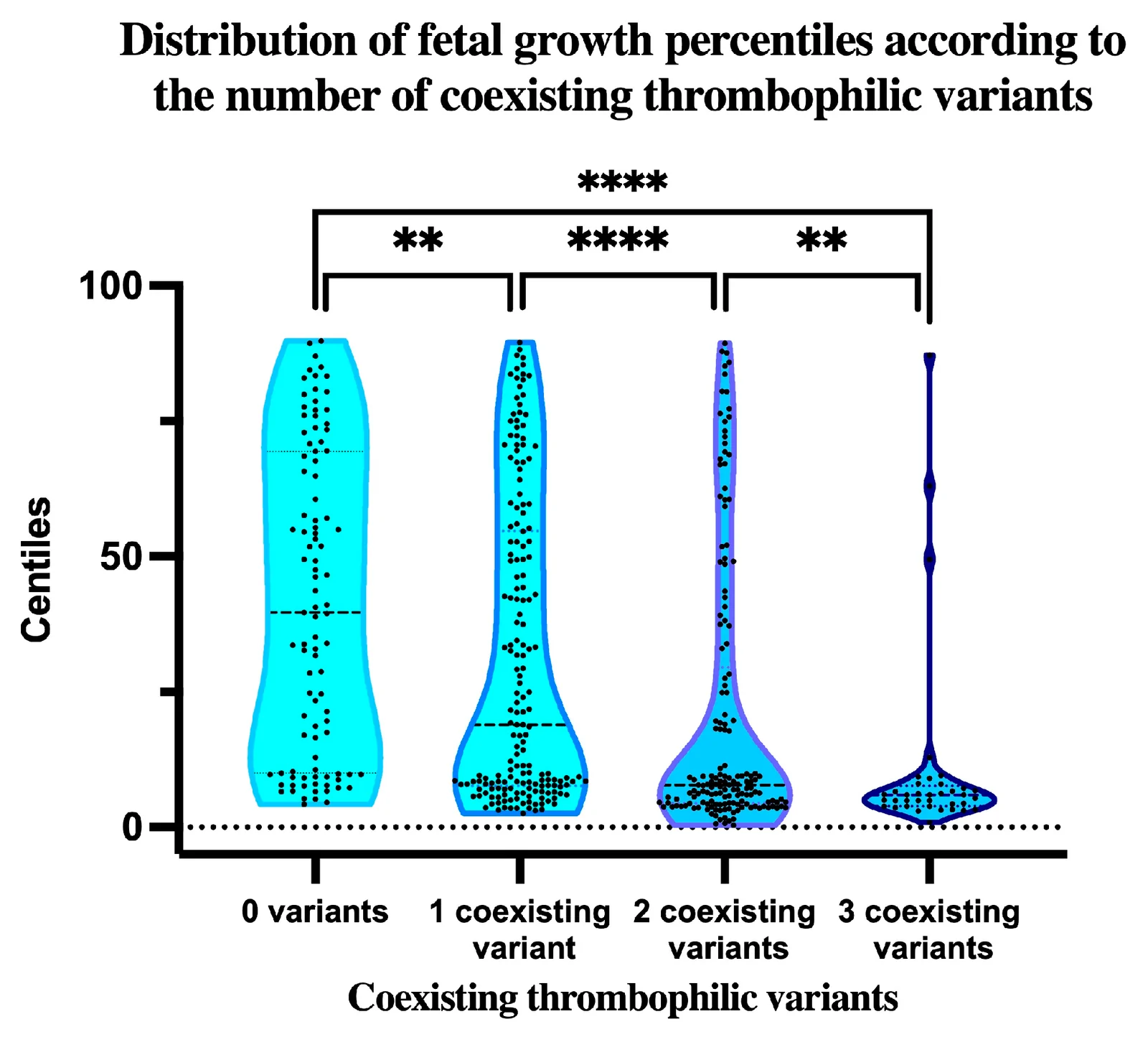
Birth-weight centile according to the number of coexisting thrombophilic variants, in the whole study population (*n* = 480; 0 variants, *n* = 99; 1 variant, *n* = 182; 2 variants, *n* = 162; 3 variants, *n* = 37). Violin plots display the full distribution; the dashed line marks the median and the dotted lines the quartiles. Points represent individual patients. Kruskal–Wallis H = 79.90, *p* < 0.001, asterisks indicate significant pairwise differences between groups (Kruskal–Wallis test followed by Dunn’s multiple comparisons test): ** *p* < 0.01; **** *p* < 0.0001; exact values for all six pairwise comparisons are given in Supplementary Table S8. Because the analysis pools cases and controls, part of the gradient reflects the case–control design and the figure should be read as descriptive; the within-case analysis reported in Section 3.4 is the inferentially meaningful one.

The number of thrombophilic variants correlated inversely with birth weight centile (Spearman coefficient ρ = −0.406; *p* < 0.001; Figure 6). Because this analysis pools cases and controls, and case status was itself defined by centile, part of this correlation is imposed by the sampling scheme. It was therefore repeated within the case group alone: among the 240 growth-restricted pregnancies the correlation persisted (Spearman ρ = −0.315; *p* < 0.001), with mean birth-weight centile falling from 7.8 in women carrying no variant to 6.7, 5.6 and 5.4 in those carrying one, two and three variants (Kruskal–Wallis H = 26.2; *p* < 0.001). Within the control group no such correlation was present (ρ = −0.041; *p* = 0.53). Exact post hoc comparisons are given in Supplementary Table S8. The mean centile progressively decreased from 41.4 in patients without any variants to 31.3, 21.0, and 10.6 in those with one, two, and three variants, respectively, and the mean birth weight decreased from 2995 ± 760 g to 2704 ± 915 g, 2356 ± 893 g, and 2133 ± 685 g (Kruskal–Wallis test *p* < 0.001). The first step of this gradient—one variant against none—does not survive Bonferroni adjustment across all 36 comparisons (Bonferroni-adjusted *p* = 0.228), although it survives the Benjamini–Hochberg procedure (q = 0.008); the estimates for two and for three variants survive both (Supplementary Table S7d).

### 3.5. Multivariate Analysis

In multiple linear regression analysis with weight centile as the dependent variable, the presence of the PAI-1 4G allele was associated with a 12.34 centile point reduction (95% CI −17.20–−7.49; *p* < 0.001), factor V Leiden with a 10.23 point reduction (95% CI −15.96–−4.50; *p* < 0.001), and the *MTHFR* 677T allele with a 8.05 point reduction (95% CI −12.80–−3.29; *p* < 0.001).

**Figure 7:**
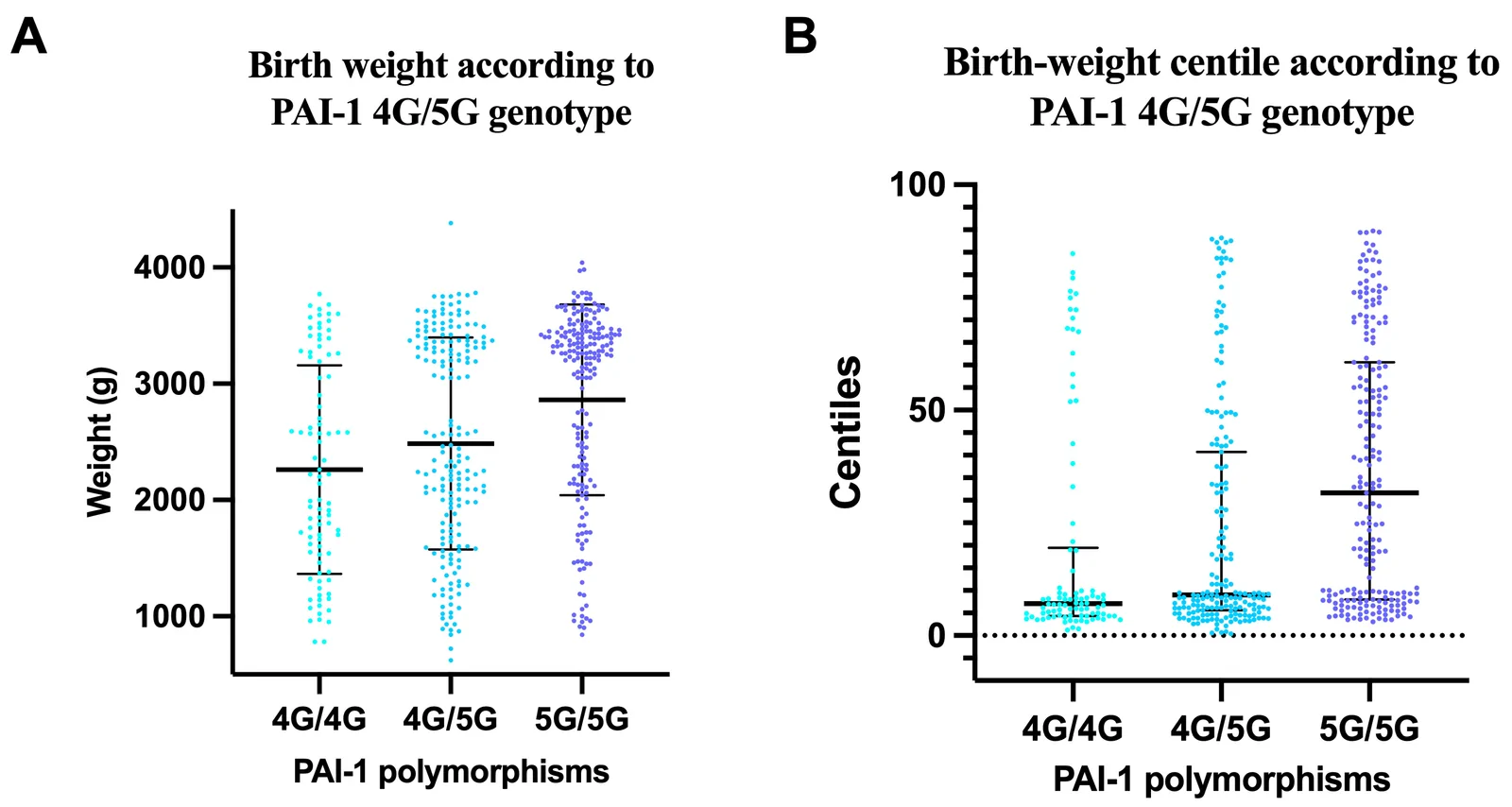
Birth weight (**A**) and birth weight centile (**B**) according to PAI-1 4G/5G genotype, in the whole study population (4G/4G, *n* = 90; 4G/5G, *n* = 187; 5G/5G, *n* = 203). Points represent individual patients; horizontal lines show the mean and standard deviation in panel (**A**), and the median with interquartile range in panel (**B**). Mean birth weight was 2262 ± 897 g, 2485 ± 912 g and 2862 ± 820 g for the 4G/4G, 4G/5G and 5G/5G genotypes, respectively (one-way ANOVA, *p* < 0.001). Median birth weight centile was 7.1 (interquartile range 4.4–19.0), 9.0 (5.6–39.1) and 31.7 (8.2–60.6), respectively (Kruskal–Wallis test, *p* < 0.001).

The effect of PAI-1 genotype on birth weight and centile is illustrated in Figure 7. Mean birth weight decreased from 2862 ± 820 g in patients with genotype 5G/5G to 2485 ± 912 g in those with genotype 4G/5G and to 2262 ± 897 g in those with genotype 4G/4G (one-way ANOVA *p* < 0.001), with a corresponding decrease in mean centile from 36.3 ± 28.5 to 23.8 ± 26.3 and 19.6 ± 25.2, respectively (*p* < 0.001). We report the effect of genotype on birth-weight centile rather than on absolute birth weight adjusted for gestational age. Birth-weight centile is standardised for gestational age by construction, whereas a model of absolute weight that conditions on gestational age at delivery conditions on a variable lying on the causal pathway between growth restriction and iatrogenic preterm birth, so that its coefficients do not have a straightforward interpretation. In the unadjusted birth-weight scale, and for context only, carriage of the PAI-1 4G allele was associated with a birth weight lower by 392 g (95% CI −548 to −235), factor V Leiden by 382 g (−567 to −197) and *MTHFR* 677T by 147 g (−300 to +7); these estimates reflect both reduced growth and earlier delivery and are not presented as independent effects on fetal growth.

**Figure 8:**
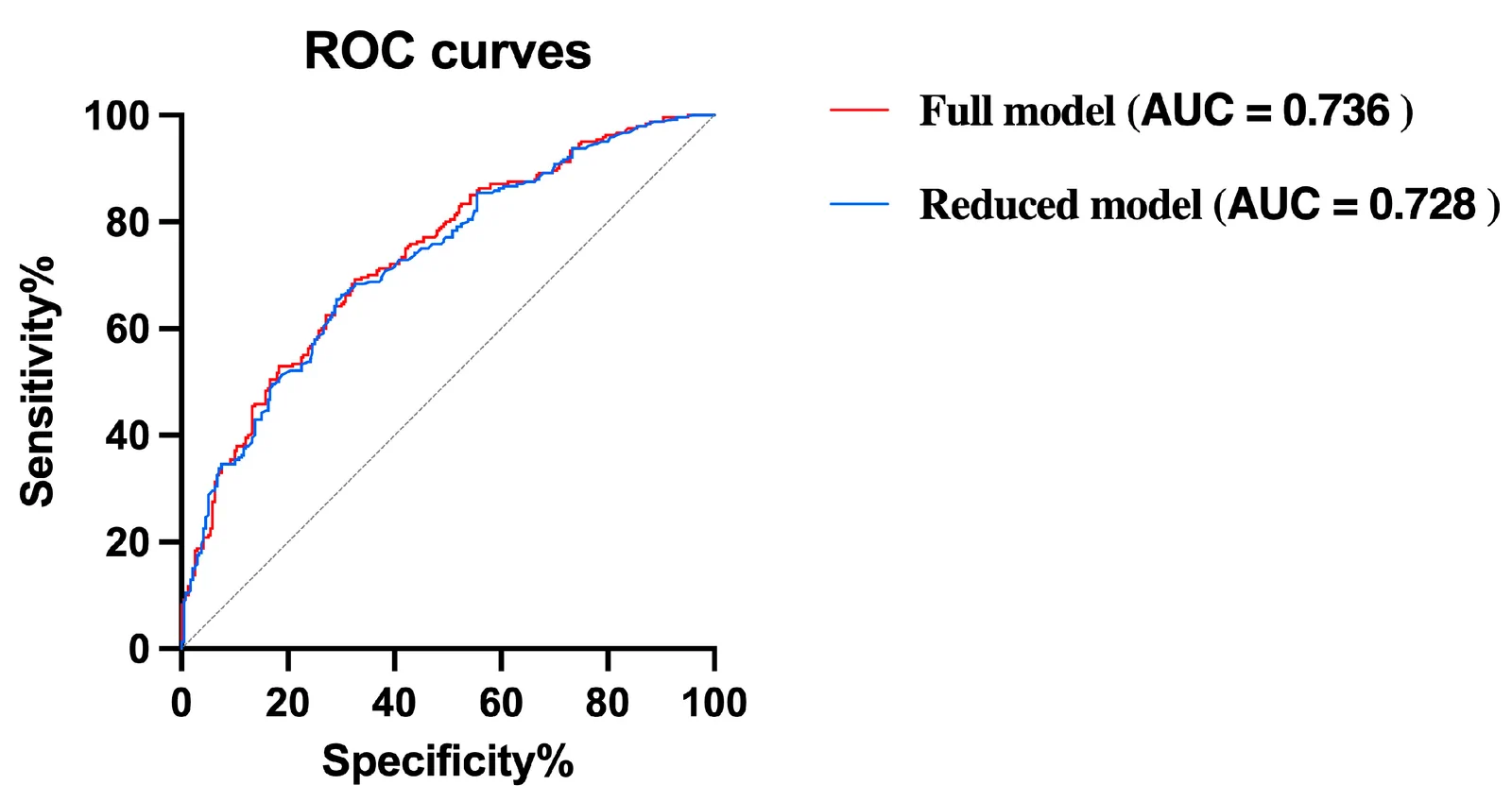
Receiver operating characteristic (ROC) curves of the logistic regression models for the prediction of fetal growth restriction. The extended model includes the three thrombophilic polymorphisms and all maternal covariates (area under the curve 0.736); the reduced model includes only the three polymorphisms and pre-gestational body mass index (area under the curve 0.728). The dashed diagonal represents the line of no discrimination.

In the multivariable logistic regression model, adjusted for maternal age, pregestational body mass index, smoking, parity, chronic hypertension, pregestational diabetes mellitus, and history of hypertensive disease in pregnancy, all three polymorphisms remained independent predictors of fetal growth restriction (Figure 3B): factor V Leiden (aOR 3.33; 95% CI 2.03–5.47; *p* < 0.001), PAI-1 4G (aOR 2.83; 95% CI 1.89–4.24; *p* < 0.001), and *MTHFR* 677T (aOR 2.00; 95% CI 1.35–2.97; *p* < 0.001). Body mass index also remained an independent predictor (aOR 1.15 per unit; 95% CI 1.06–1.25; *p* = 0.001), while maternal age, smoking, parity, and comorbidities did not reach statistical significance. The model had moderate discriminative performance (area under the ROC curve = 0.736; McFadden pseudo-R^2^ = 0.131; Nagelkerke pseudo-R^2^ = 0.221; Figure 8). The model was well calibrated (Hosmer–Lemeshow χ^2^ = 9.69; df = 8; *p* = 0.288; Brier score 0.208), variance inflation factors were all below 1.20, the linearity assumption in the logit held for maternal age and body mass index, and no influential observation altered the estimates; with 240 events and 10 covariates the model had 24 events per variable. Bootstrap internal validation with 1000 replications gave an optimism-corrected area under the curve of 0.713. Removing the three genotypes from the model reduced the area under the curve from 0.736 to 0.614, and a likelihood ratio test for the genotype block was highly significant (χ^2^ = 72.2; df = 3; *p* < 0.001), indicating that genotype contributes independently of the maternal covariates. Because prediction was not an aim of this study, the ROC analysis should be regarded as exploratory. Full diagnostics are given in Supplementary Table S4 and the calibration plot in Supplementary Figure S1. The results of the multivariable analyses are presented in Table 5 and Table 6.

Pairwise interaction terms between the three polymorphisms were added to the model. A positive interaction was observed between the PAI-1 4G and factor V Leiden alleles (interaction OR 3.28; *p* = 0.026) and the three terms were jointly significant at the conventional threshold (likelihood ratio *p* = 0.045), but neither result survives adjustment for the three interaction tests performed (Bonferroni-adjusted *p* = 0.078). The data are therefore compatible with, but do not establish, a supra-additive effect of coexisting PAI-1 4G and factor V Leiden alleles.

R^2^ = 0.107. Birth-weight centile is standardised for gestational age by construction; we therefore model it directly rather than modelling absolute birth weight with adjustment for gestational age, which lies on the causal pathway between growth restriction and iatrogenic preterm birth. In a model additionally adjusted for pre-gestational body mass index, maternal age, smoking and parity the estimates were essentially unchanged (PAI-1 4G −12.31, 95% CI −17.16 to −7.46; *F5* A −10.34, −16.10 to −4.58; *MTHFR* 677T −7.71, −12.47 to −2.95; R^2^ = 0.118). CI, confidence interval. In the sensitivity analysis restricted to cases below the 5th centile, the direction of the association with pre-gestational body mass index is reversed (adjusted OR 0.81; 95% CI 0.68–0.96; Supplementary Table S2b). We draw no inference from this reversal, which is discussed in Section 4.7.

### 3.6. Perinatal Outcomes

**Figure 9:**
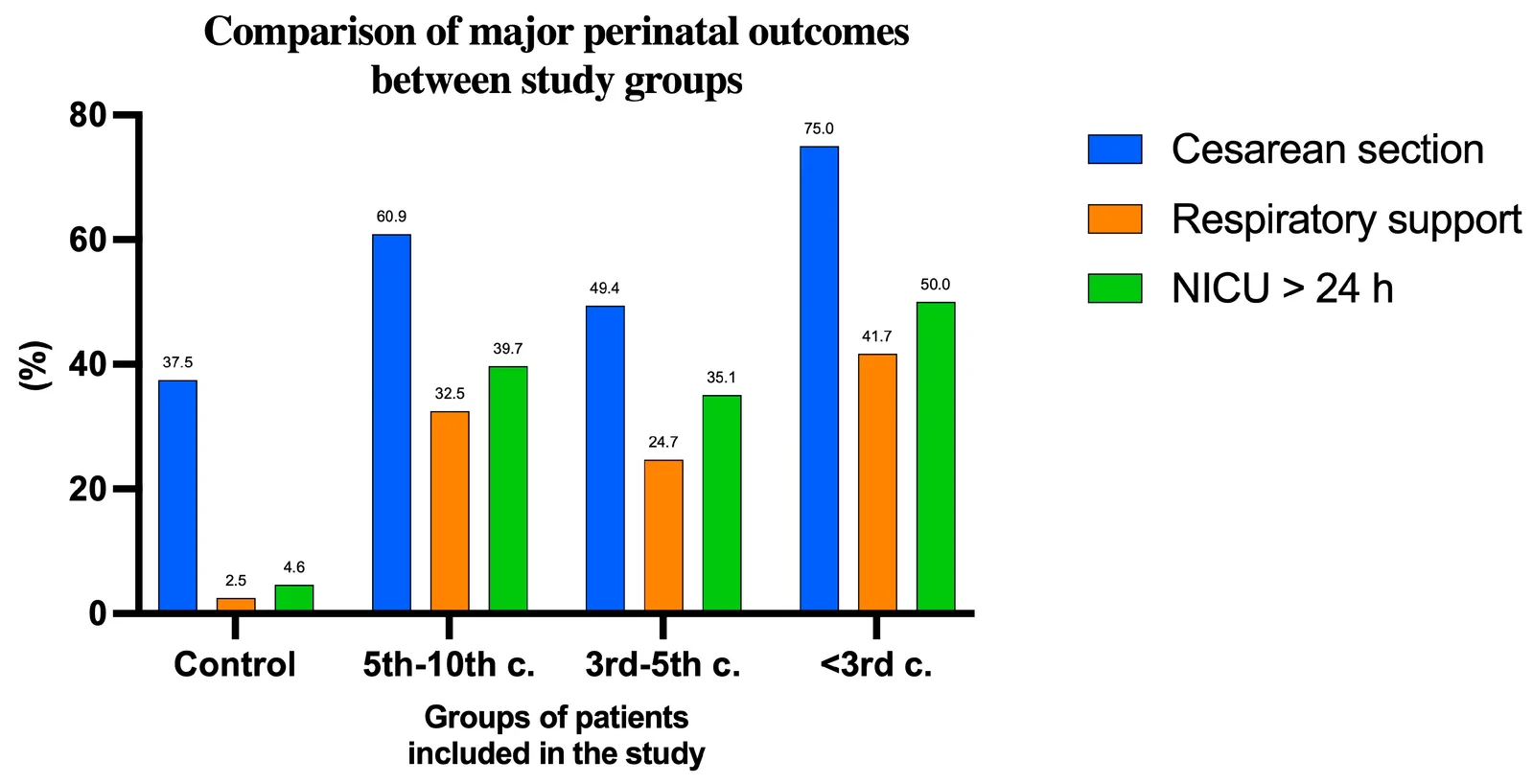
Categorical perinatal outcomes across control subjects and the three subgroups of increasing fetal growth restriction severity: caesarean section, neonatal respiratory support and admission to the neonatal intensive care unit (NICU) for more than 24 h. χ^2^ test across the four groups: *p* < 0.001 for all three outcomes. Because gestational age at delivery differs by design between controls and cases, these comparisons describe the clinical burden of the phenotype rather than an independent effect of growth restriction.

**Figure 10:**
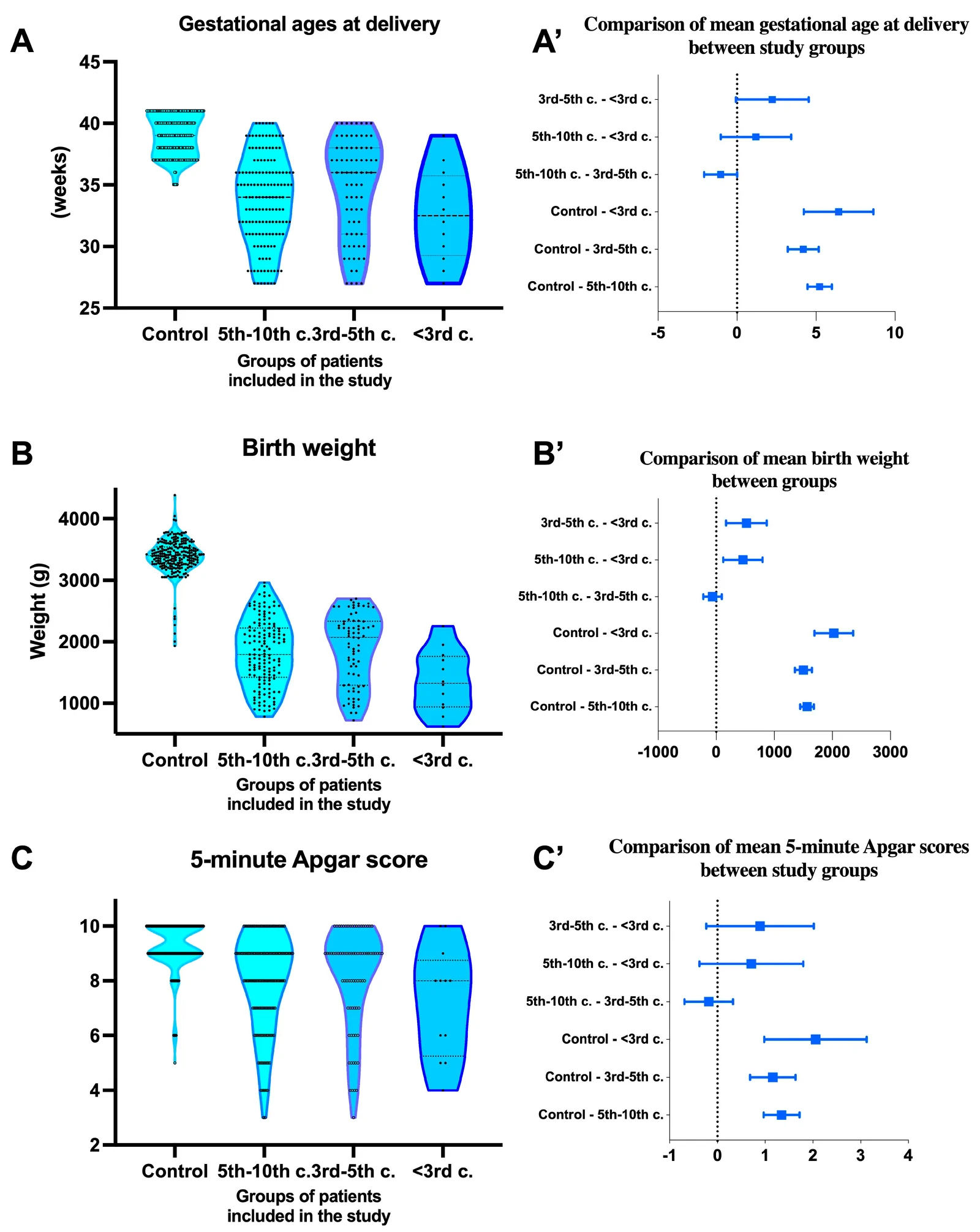
Continuous perinatal outcomes across control subjects and the three subgroups of increasing fetal growth restriction severity. (**A**) Gestational age at delivery. (**B**) Birth weight. (**C**) 5 min Apgar score. Violin plots display the full distribution; the dashed line marks the median and the dotted lines the quartiles, and points represent individual patients. The Apgar score takes only integer values, so the density estimate in panel **C** is shown for visual comparison only. Kruskal–Wallis test: *p* < 0.001 for all three variables. Panels (**A’**,**B’**,**C’**) show the corresponding pairwise mean differences between groups with 95% confidence intervals; the dotted vertical line marks a difference of zero, and intervals that do not cross it denote statistically significant differences.

Perinatal outcomes are summarized in Table 7 and Table 8 and Figure 9 and Figure 10. Because gestational age at delivery differs between the two groups by design, the comparisons of perinatal outcome in Table 7 and Table 8 are presented as descriptive of the clinical burden of the phenotype and not as independent effects of growth restriction; odds ratios have accordingly been removed from those tables.

Pregnancies complicated by fetal growth restriction ended significantly earlier (34.05 ± 3.78 vs. 39.02 ± 1.63 weeks; *p* < 0.001), with a preterm birth rate of 70.4% vs. 2.9% in the control group (*p* < 0.001) and a birth rate before 32 weeks of 27.5% vs. 0% (*p* < 0.001). The mean birth weight was 1819.21 ± 557.63 g in the growth restriction group and 3386.19 ± 287.88 g in the control group (*p* < 0.001), and the mean weight centile was 6.13 ± 2.28 versus 50.50 ± 23.75 (*p* < 0.001).

Subgroup analysis revealed similar weights in the 5–10th centile and 3–5th centile subgroups (1822.3 ± 537.3 g versus 1884.5 ± 578.7 g) and a significantly lower weight in the subgroup below the 3rd centile (1361.7 ± 498.1 g; *p* < 0.001 for the overall comparison). The absence of a decrease in absolute weight between the first two subgroups is explained by the higher gestational age at birth in the subgroup with the 3–5th centile (34.8 ± 4.0 vs. 33.8 ± 3.6 weeks), which is why the centile, and not the absolute weight, correctly reflects the severity gradient (7.6 ± 1.4 vs. 4.0 ± 0.6 vs. 1.5 ± 0.8; *p* < 0.001).

Caesarean section was performed in 57.9% of cases compared with 37.5% of controls (*p* < 0.001). The 5 min Apgar score was significantly lower in the growth-restricted group (7.98 ± 1.80 versus 9.30 ± 0.86; *p* < 0.001), and a score below 7 was recorded in 21.7% of growth-restricted newborns compared with 2.9% of controls (*p* < 0.001). Neonatal morbidity was substantially higher in the growth-restricted group: respiratory support was required in 30.4% of infants compared with 2.5% (*p* < 0.001), and admission to neonatal intensive care for more than 24 h in 38.8% compared with 4.6% (*p* < 0.001). As gestational age at birth differs by design between the two groups, these comparisons should be considered descriptive of the clinical burden of the growth-restricted phenotype, and not as independent effects of the restriction itself.

## 4. Discussion

### 4.1. Main Results

The present study of 480 pregnancies found independent and significant associations between all three thrombophilic polymorphisms studied and fetal growth restriction. The strongest adjusted association was observed for factor V Leiden (aOR 3.33; 95% CI 2.03–5.47), followed by the PAI-1 4G allele (aOR 2.83; 95% CI 1.89–4.24) and the *MTHFR* 677T allele (aOR 2.00; 95% CI 1.35–2.97). Three observations are worth highlighting: the existence of a gradient of risk depending on the severity of growth restriction, a marked cumulative effect of coexisting variants, and the maintenance of associations after adjustment for maternal confounders, with a quantitative translation into birth-weight centile.

### 4.2. PAI-1 4G/5G Polymorphism

This association identified in our cohort—an approximately threefold higher odds in carriers of the 4G allele (OR 3.02; 95% CI 2.07–4.40)—is compatible with the central role of the plasminogen activator system in placental physiology. Extravillous trophoblast invasion into the decidua and spiral arteries depends on a tightly controlled balance between plasminogen activation and inhibition; an excess of PAI-1 limits extracellular matrix degradation and restricts trophoblast invasion, while favoring fibrin deposition in the intervillous space [11,12]. The 4G allele results in significantly higher plasma levels of PAI-1, due to the absence of a transcriptional repressor binding to the promoter [13].

Our results are consistent with histopathological and biochemical observations reported by Estellés et al., who documented increased placental expression of PAI-1 in pregnant women with preeclampsia and fetal growth restriction [14], and with the data of Souza et al., who identified the 4G/4G genotype as an independent risk factor for placental insufficiency with fetal hemodynamic centralization [16]. Agersnap et al.’s systematic review of the role of PAI-1 in placental complications concluded that, of the thrombophilic variants studied, the 4G/5G polymorphism is the one for which the data are most consistent [15]. Dedicated meta-analyses have reported significant associations between the 4G/4G genotype and preeclampsia [41,42] and between PAI-1 variants and recurrent miscarriage [43,44], and Glueck et al. have described the 4G/4G genotype as an independent risk factor for severe pregnancy complications [45]. Our observation of a risk gradient—from an OR of 2.37 in the 5th–10th centile stratum to 4.07 in the 3rd–5th centile stratum and a prevalence of 100% below the 3rd centile (trend *p* < 0.001)—is compatible with an effect of PAI-1 that increases with the degree of placental compromise. The reduction of 12.3 birth-weight centile points in carriers of the 4G allele provides a quantitative measure of this association.

### 4.3. MTHFR C677T Polymorphism

The association observed for *MTHFR* C677T was the weakest of the three. Both the heterozygous and the homozygous genotypes pointed in the same direction (OR 2.11 and 1.86 versus CC, respectively), but the estimate for the TT genotype did not reach statistical significance (95% CI 0.93–3.74; *p* = 0.109), most plausibly because only 37 women in the entire cohort carried it. Post hoc power was 42% for this comparison and 14% for the recessive model, so the study is underpowered at this locus and the absence of a demonstrable gene-dosage effect should not be read as evidence against one. This observation is part of a broader context of skepticism about the clinical relevance of *MTHFR* genotyping. Although hyperhomocysteinemia is associated with endothelial dysfunction and placental vasculopathy [19,20], and some studies have reported associations between the C677T variant and low birth weight or preeclampsia [46,47], the effect of the variant on homocysteinemia is largely conditioned by folic acid status, which in populations with adequate intake or food fortification is sufficient to cancel the functional difference between genotypes [17,18]. Added to this is the marked geographic and ethnic variability of the frequency of the 677T allele, documented in over 7000 newborns from 16 regions of the world [48], which makes the risk estimates obtained in one population not directly transferable to another; moreover, meta-analyses have not confirmed a consistent effect of this variant for recurrent miscarriage either [49]. The American College of Medical Genetics practice guidelines explicitly recommend against testing for *MTHFR* polymorphisms in the evaluation of thrombophilia [32], a position recently reaffirmed by a joint call from several thrombosis and hemostasis societies [33]. Periconceptional folic acid supplementation remains universally recommended, regardless of genotype, and has been associated with increased fetal weight in large cohort studies [50]. Therefore, we believe that our *MTHFR* result should be interpreted as a marker of association in a specific population rather than as an argument for routine testing.

In the Doppler-characterised series from the same department, carriage of the MTHFR 677T allele was not associated with abnormal uteroplacental Doppler (OR 0.50) or with third-trimester notching (OR 0.44), in contrast to factor V Leiden and PAI-1 (Supplementary Table S9b). The series is small and none of these estimates reaches the conventional threshold, but the Doppler data do not support a placental-vascular mechanism for MTHFR in the way they do for the other two variants. This is a further reason to interpret the MTHFR association as a marker in a specific population rather than as evidence of a mechanism.

### 4.4. Factor V Leiden

The association between factor V Leiden and growth restriction identified in our cohort (OR 3.64; 95% CI 2.27–5.83) is somewhat stronger than the pooled estimate reported by meta-analyses of case–control studies. The TREATS systematic review, which included 79 studies, reported an OR of 2.68 for the association between heterozygous factor V Leiden and fetal growth restriction [23]. Activated protein C resistance determined by the Arg506Gln substitution favors thrombus formation in the uteroplacental circulation and perivillous fibrin deposition, lesions characteristic of maternal vascular malperfusion [8,21].

However, it should be emphasized that this association has not been confirmed by prospective studies. The meta-analysis of prospective cohort studies by Rodger et al. did not show a significant association between factor V Leiden and placental complications [24], and the prospective cohorts by Said et al. and the NICHD network reached similar conclusions [25,26]. The discrepancy is mainly attributed to selection and publication bias in retrospective case–control studies, a category to which the present study belongs. In addition, the randomized trials TIPPS, FRUIT, and ALIFE failed to demonstrate a benefit of low-molecular-weight heparin prophylaxis on placental complications in pregnant women with hereditary thrombophilia [27,28,29], leading current guidelines to not recommend routine testing [30,31,51].

A notable feature of our results is the graded relationship between factor V Leiden carriage and the severity of growth restriction. Carrier prevalence rose from 12.1% in controls to 22.5% in the 5th–10th centile subgroup (OR 2.11; 95% CI 1.23–3.64; *p* = 0.007), 48.1% in the 3rd–5th centile subgroup (OR 6.73; 95% CI 3.72–12.17) and 75.0% in the subgroup below the 3rd centile (OR 21.83; 95% CI 5.58–85.31), the odds ratio approximately doubling at each successive step (Cochran–Armitage trend *p* < 0.001). Of the three variants studied, factor V Leiden showed the steepest severity gradient, which is compatible with a contribution of uteroplacental thrombosis that increases across the phenotypic spectrum. The association is nevertheless already detectable in the mildest stratum, so these data do not support the notion that factor V Leiden contributes exclusively to severe restriction. The estimate in the subgroup below the 3rd centile rests on 12 patients and its confidence interval is correspondingly wide.

### 4.5. Cumulative Effect of Thrombophilias

One of the most consistent observations of the study is the graded increase in the odds of growth restriction with the number of coexisting thrombophilic variants, from an OR of 2.12 (95% CI 1.24–3.64) for one variant to 5.60 (3.21–9.78) for two and 24.42 (7.87–75.78) for all three, the last resting on 33 cases and only 4 controls and therefore imprecise. The inverse correlation between the number of variants and birth-weight centile (ρ = −0.406 overall; ρ = −0.315 within the case group alone) is consistent with a graded association with variant load, in which each variant contributes a modest individual effect, but their coexistence generates a prothrombotic and hypofibrinolytic phenotype with significant clinical impact. This observation is consistent with previous data suggesting an additive effect of combined thrombophilias on placental complications [23,52] and offers a possible explanation for the inconsistency of the literature: studies that analyze a single polymorphism in isolation may underestimate the risk in patients with multiple genotypes. We describe this as a graded association with variant load rather than as a demonstrated cumulative biological effect. Formal testing of the three pairwise interaction terms did not establish departure from additivity on the log-odds scale after adjustment for the number of tests performed, and the analysis pooling cases and controls is partly structured by the sampling design; the within-case analysis reported in Section 3.4 is the inferentially meaningful one.

### 4.6. Clinical Implications

Our results do not, by themselves, justify universal screening for thrombophilia in pregnant women with FGR, a position that remains in line with current guidelines [30,31]. Published data from the same geographic area show, moreover, that the main determinants of neonatal hospitalization duration are birth weight centile, Apgar score, and gestational age, and not absolute birth weight [35]—an observation that supports our choice to use centile as a measure of severity and to report perinatal outcomes separately by centile subgroups. Whether the coexistence of several polymorphisms identifies a subgroup that would benefit from more intensive ultrasound and Doppler surveillance is a hypothesis raised by these data, not a recommendation supported by them. The present design—retrospective, single-centre, with a genetically tested and therefore selected control group, a biometrically defined phenotype without Doppler confirmation, an unusual control genotype frequency at one locus and no external validation—cannot establish that genotype-guided surveillance would improve prediction or outcome, and we make no such recommendation. Low-dose aspirin administration starting before 16 weeks of gestation in pregnant women at high risk of placental complications remains the only preventive intervention with demonstrated benefit in randomized trials [1,53], and anticoagulant prophylaxis based solely on genotype is currently unsupported by evidence.

### 4.7. Limitations

First, the retrospective case–control design is vulnerable to selection bias and does not allow for the establishment of a causal relationship. As shown, meta-analyses of prospective studies have not confirmed the associations reported by case–control studies [24,25,26], and our results should be interpreted with caution in this context as well. Inclusion required complete genotyping for all three polymorphisms, and the clinical indication for ordering a thrombophilia panel is unlikely to have been the same in a pregnancy complicated by growth restriction and in one with normal growth. If testing among controls was preferentially requested for women with a personal or family history suggestive of thrombophilia, the control group is enriched for carriers and our odds ratios are biased towards the null; if testing was near-universal among cases and sporadic among controls, the bias could operate in the opposite direction. The magnitude of the associations we report should be regarded as uncertain for this reason. The comparisons across severity strata, which are made within the case group, are not affected by differential testing between cases and controls and constitute the more robust element of our findings.

Second, although genotype distributions in the control group conformed to Hardy–Weinberg equilibrium for *MTHFR* C677T (exact *p* = 0.205) and *F5* G1691A (exact *p* = 1.000), a significant departure persisted for PAI-1 4G/5G (exact *p* = 0.016), taking the form of a deficit of heterozygotes (fixation index 0.158). We consider a technical explanation the most plausible. Genotyping was performed in the course of clinical care in several external laboratories rather than under a single standardised protocol, and the 4G/5G variant is a single-base insertion/deletion at which incomplete discrimination of heterozygotes is a recognised difficulty and is likely to differ between assays; a departure confined to this locus is what between-laboratory heterogeneity in allelic discrimination would be expected to produce. Population stratification is unlikely, since it would be expected to affect all three loci, whereas the other two are in equilibrium in both groups. Two observations limit the consequences of this. Laboratory allocation followed the referral pathway and not the outcome of the pregnancy, so any resulting misclassification is expected to be non-differential and to bias the associations towards the null. And restricting the PAI-1 comparison to the two homozygous genotypes, which are unaffected by heterozygote miscalling, left the association undiminished (4G/4G vs. 5G/5G: OR 4.68; 95% CI 2.73–8.03). The carrier prevalence of factor V Leiden in our control group was 12.1%, corresponding to an allele frequency of 6.0%, which lies at the upper end of the 2–7% range reported for European populations and is consistent with the higher frequencies described in south-eastern Europe; the observed carrier prevalence is what the observed allele frequency predicts under Hardy–Weinberg equilibrium (11.7% expected). A control group drawn from pregnancies tested for thrombophilia on clinical indication is nevertheless likely to be enriched for carriers relative to the general obstetric population, which would bias our odds ratios towards the null. Third, and most importantly, the phenotype was defined biometrically, without Doppler indices of the umbilical artery, middle cerebral artery, cerebroplacental ratio or uterine arteries, and cannot therefore distinguish placentally mediated growth restriction from a constitutionally small fetus. In a Doppler-characterised series of 80 pregnancies from the same department, using the same severity strata, uterine and umbilical artery indices in the 5th–10th centile stratum were indistinguishable from controls (any uteroplacental abnormality 2/25 versus 3/40; *p* = 1.00), whereas in the strata below the 5th centile they were clearly abnormal (11/15 versus 3/40; OR 33.9; *p* < 0.001). The mildest stratum, which contains 63% of our cases, is therefore likely to be substantially enriched for constitutionally small fetuses. Such misclassification is non-differential with respect to maternal genotype and biases the odds ratios towards the null. Consistent with this, restricting the case definition to the 89 pregnancies below the 5th centile strengthened every association (PAI-1 4G, OR 4.90; *MTHFR* 677T, OR 2.40; factor V Leiden, OR 7.78; adjusted ORs 5.69, 3.24 and 8.37 respectively; Supplementary Table S2). Mechanistic interpretation should nevertheless be confined to the strata below the 5th centile, and the estimates for the cohort as a whole should be read as pertaining to a small-for-gestational-age phenotype. The reversal of the body mass index coefficient in the restricted model deserves a word. Maternal adiposity is associated with higher absolute birth weight, so restricting the case group to the most severely growth-restricted pregnancies is expected to deplete it of women with higher body mass index; the two models are fitted to different case sets and the restricted one contains 89 cases. We report the reversal for completeness and draw no causal inference from it. Fourth, hypertensive disease of the index pregnancy was not recorded in the research database, and this is a potentially important confounder, since preeclampsia and placentally mediated growth restriction share the same substrate of maternal vascular malperfusion. To quantify how influential such an unmeasured confounder would have to be, we computed E-values for the adjusted associations: a confounder would have to be associated with both the genotype and growth restriction by a risk ratio of at least 6.12 for factor V Leiden, 5.11 for PAI-1 4G and 3.41 for *MTHFR* 677T—and of at least 3.48, 3.19 and 2.04 respectively to move the confidence intervals to include the null—over and above the measured covariates (Supplementary Table S10). Reported associations between hypertensive disease of pregnancy and these polymorphisms are of the order of 1.2–1.8, and between hypertensive disease and growth restriction of the order of 2–3. Both conditions must be met simultaneously for a confounder to explain an association, and the exposure-side association is the smaller of the two, so preeclampsia is unlikely on its own to account for our findings. The margin is narrowest for *MTHFR* 677T, whose E-value for the confidence limit is 2.04 and therefore lies within the range reported for the confounder–outcome association; the *MTHFR* association is accordingly the least robust of the three to unmeasured confounding by hypertensive disease. This analysis does not replace measurement of the variable. Data on placental histopathology, plasma PAI-1 and homocysteine, folate status, the angiogenic markers sFlt-1 and PlGF, and antiplatelet or anticoagulant treatment were likewise not available.

Fifth, genotyping was performed in the course of clinical care, in several external laboratories accredited by the national health authority, rather than under a single research protocol. We are consequently unable to report assay-level information—extraction kits, instruments, assay identifiers, reaction conditions, plate-level positive and no-template controls, first-pass call rates and DNA quality metrics—and unable to exclude between-laboratory differences in allelic discrimination. We regard this as the most plausible explanation for the departure from Hardy–Weinberg equilibrium at PAI-1 4G/5G described above. Independent validation of a blinded random subset of samples by a second method was not performed. We regard it as the appropriate next step and it is a defined priority of the prospective extension of this study, together with prospective recording of DNA quantification and quality metrics; its absence is an outstanding limitation of the present work. Independent validation by sequencing, in a single laboratory and under a research protocol, is a defined priority of the prospective extension of this study, together with prospective recording of DNA quantification and quality metrics.

Another major limitation was that the severely restricted subgroup (below the 3rd centile) included only 12 patients, which severely limits the precision of the estimates in this subgroup; the low prevalence of *MTHFR* 677T carriers in this subgroup (3 of 12; 25.0%) most likely has a sampling explanation and should not be read as a protective effect.

Finally, the study is monocentric and included an ethnically homogeneous population, which limits the generalizability of the results.

The Doppler-characterised series and the comparison of effect sizes under the two case definitions are presented in Supplementary Table S9 and Supplementary Figure S2.

### 4.8. Future Research Directions

Confirmation of these results requires prospective studies that include complete Doppler assessment according to consensus criteria, standardised placental histopathological examination according to the Amsterdam consensus criteria [8]. This prospective extension is planned in collaboration with our institutional pathology department, whose published work has applied exhaustive immunohistochemical panels to lesions in which conventional morphology alone is not diagnostic [54]; that work is cited here as documentation of local methodological capacity and not as evidence concerning placental pathology. Confirmation will also require measurement of circulating PAI-1 and homocysteine levels and their correlation with genotype, as well as systematic recording of pharmacological interventions. Integrating thrombophilic genotype into multivariable prediction models, together with biochemical markers and Doppler of the uterine arteries in the first trimester, could improve early identification of pregnancies at risk of placental insufficiency. Independent validation of genotyping by sequencing, and estimation of the factor V Leiden allele frequency in a population-representative regional sample, are further priorities arising from the present work.

## 5. Conclusions

In this cohort, the PAI-1 4G/5G, *MTHFR* C677T and factor V Leiden polymorphisms were independently associated with a small-for-gestational-age phenotype, the strongest adjusted association being that of factor V Leiden. The odds increased with the severity of restriction—significantly so for all three variants across in the two case strata above the 3rd centile, and most steeply for factor V Leiden—and with the number of coexisting variants reaching an odds ratio of 24.42 in the small group of 33 cases and 4 controls carrying all three. These associations were maintained after adjustment for maternal confounders and were quantitatively reflected in a significant reduction in birth-weight centile. Affected pregnancies had substantially higher perinatal morbidity, with increased rates of prematurity, low Apgar scores, need for respiratory support, and admission to neonatal intensive care.

The results are compatible with a contribution of hypofibrinolysis and an inherited prothrombotic state to the placental insufficiency phenotype, most clearly in the strata below the 5th centile, in which placental Doppler abnormality is demonstrable, and they do not establish a causal mechanism. However, they do not justify, at the current state of evidence, routine screening for hereditary thrombophilia in pregnant women with fetal growth restriction, nor anticoagulant prophylaxis based solely on genotype. Prospective validation, including Doppler assessment and placental histopathology, is necessary before any change in clinical practice.

## Figures and Tables

**Table 1:** Maternal characteristics of the study groups.

Characteristic	FGR (*n* = 240)	Control (*n* = 240)	*p*
Maternal age, years	26.40 ± 4.38	26.22 ± 4.12	0.550
Pre-gestational BMI, kg/m^2^	23.94 ± 2.65	23.26 ± 2.32	**0.002**
Multiparity, *n* (%)	66 (27.5)	71 (29.6)	0.686
Smoking, *n* (%)	13 (5.4)	11 (4.6)	0.835
Pre-gestational diabetes, *n* (%)	13 (5.4)	13 (5.4)	1.000
Chronic hypertension, *n* (%)	5 (2.1)	5 (2.1)	1.000
Autoimmune disease, *n* (%)	2 (0.8)	4 (1.7)	0.686
Prior hypertensive disease of pregnancy, *n* (%)	14 (5.8)	5 (2.1)	0.058
Gestational diabetes, *n* (%)	3 (1.2)	12 (5.0)	**0.032**

Data are presented as mean ± standard deviation or as absolute frequency (percentage). Continuous variables were compared using the Mann–Whitney test and categorical variables using Fisher’s exact test. BMI, body mass index; FGR, fetal growth restriction. Statistically significant *p* values are shown in bold.

**Table 2:** Genotype and allele distribution in the FGR group and the control group.

Polymorphism/Genetic Model	FGR (*n* = 240) *n* (%)	Control (*n* = 240) *n* (%)	OR (95% CI)	*p*	q (BH)
PAI-1 4G/5G (rs1799889)					
5G/5G (reference)	70 (29.2)	133 (55.4)	1.00	–	
4G/5G	106 (44.2)	81 (33.8)	2.49 (1.65–3.74)	<0.001	2.10 × 10^−5^
4G/4G	64 (26.7)	26 (10.8)	4.68 (2.73–8.03)	<0.001	3.83 × 10^−8^
4G carrier (dominant model)	170 (70.8)	107 (44.6)	3.02 (2.07–4.40)	<0.001	3.83 × 10^−8^
4G/4G vs. 4G/5G + 5G/5G (recessive model)	64 (26.7)	26 (10.8)	2.99 (1.82–4.92)	<0.001	2.10 × 10^−5^
4G allele (allelic model), *n*/480 (%)	234 (48.8)	133 (27.7)	2.48 (1.90–3.25)	<0.001	3.51 × 10^−10^
*MTHFR* C677T (rs1801133)					
CC (reference)	103 (42.9)	146 (60.8)	1.00	–	
CT	116 (48.3)	78 (32.5)	2.11 (1.44–3.09)	<0.001	1.80 × 10^−4^
TT	21 (8.8)	16 (6.7)	1.86 (0.93–3.74)	0.109	0.127
677T carrier (dominant model)	137 (57.1)	94 (39.2)	2.07 (1.44–2.97)	<0.001	1.80 × 10^−4^
TT vs. CT + CC (recessive model)	21 (8.8)	16 (6.7)	1.34 (0.68–2.64)	0.494	0.494
677T allele (allelic model), *n*/480 (%)	158 (32.9)	110 (22.9)	1.65 (1.24–2.20)	<0.001	8.97 × 10^−4^
Factor V Leiden G1691A (rs6025)					
GG (reference)	160 (66.7)	211 (87.9)	1.00	–	
GA	79 (32.9)	29 (12.1)	3.59 (2.24–5.76)	<0.001	9.13 × 10^−8^
AA	1 (0.4)	0 (0.0)	3.95 (0.16–97.69)	0.433	0.466
A allele carrier (dominant model)	80 (33.3)	29 (12.1)	3.64 (2.27–5.83)	<0.001	9.13 × 10^−8^
A allele (allelic model), *n*/480 (%)	81 (16.9)	29 (6.0)	3.16 (2.02–4.93)	<0.001	3.48 × 10^−7^

OR, odds ratio; 95% CI, 95% confidence interval calculated by the Baptista–Pike method. Allele frequencies were calculated on a total of 480 alleles per group. *p* values were obtained using Fisher’s exact test. Hardy–Weinberg equilibrium, exact *p* values: PAI-1 4G/5G—cases 0.072, controls 0.016; *MTHFR* C677T—cases 0.188, controls 0.205; *F5* G1691A—cases 0.005, controls 1.000. Full statistics, including χ^2^ values and fixation indices, are given in Supplementary Table S3. q, Benjamini–Hochberg false discovery rate applied within this family of tests; Bonferroni-adjusted *p* values for all 36 comparisons are given in Supplementary Table S7.

**Table 3:** Prevalence of thrombophilic polymorphisms by severity of growth restriction.

Polymorphism	Control (*n* = 240)	5th–10th Centile (*n* = 151)	q (BH)	3rd–5th Centile (*n* = 77)	q (BH)	<3rd Centile (*n* = 12)	q (BH)	*p* (χ^2^)	*p* (Trend) ^a^
PAI-1 4G carrier, *n* (%)	107 (44.6)	99 (65.6)	1.81 × 10^−4^	59 (76.6)	6.53 × 10^−6^	12 (100.0)	1.94 × 10^−4^	<0.001	<0.001
OR (95% CI) vs. control	1.00	2.37 (1.55–3.61)		4.07 (2.27–7.32)		n.c.			
*p* vs. control	–	<0.001		<0.001		<0.001			
PAI-1 4G/4G, *n* (%)	26 (10.8)	35 (23.2)	0.003	26 (33.8)	4.05 × 10^−5^	3 (25.0)	0.170	<0.001	<0.001
OR (95% CI) vs. control	1.00	2.48 (1.42–4.33)		4.20 (2.25–7.83)		2.74 (0.70–10.78)			
*p* vs. control	–	0.002		<0.001		0.148			
*MTHFR* 677T carrier, *n* (%)	94 (39.2)	83 (55.0)	0.004	51 (66.2)	1.22 × 10^−4^	3 (25.0)	0.407	<0.001	<0.001
OR (95% CI) vs. control	1.00	1.90 (1.26–2.86)		3.05 (1.78–5.22)		0.52 (0.14–1.96)			
*p* vs. control	–	0.003		<0.001		0.380			
*MTHFR* 677TT, *n* (%)	16 (6.7)	19 (12.6)	0.092	1 (1.3)	0.103	1 (8.3)	0.576	0.003	0.634
Factor V Leiden carrier, *n* (%)	29 (12.1)	34 (22.5)	0.011	37 (48.1)	3.16 × 10^−9^	9 (75.0)	1.30 × 10^−5^	<0.001	<0.001
OR (95% CI) vs. control	1.00	2.11 (1.23–3.64)		6.73 (3.72–12.17)		21.83 (5.58–85.31)			
*p* vs. control	–	0.007		<0.001		<0.001			

^a^ Cochran–Armitage test for trend across the four ordered severity categories. Confidence intervals were calculated by the Baptista–Pike method and *p* values using Fisher’s exact test. n.e., not estimable: all 12 pregnancies in this subgroup carried the variant, so the odds ratio has no finite estimate; the exact *p* value is computable and is reported. With a Haldane–Anscombe correction of 0.5 the odds ratio would be 31.05 (95% CI 1.82–530.40), a value given in Supplementary Table S7b only to indicate direction and imprecision. q, Benjamini–Hochberg false discovery rate applied within this family of tests; Bonferroni-adjusted *p* values for all 36 comparisons are given in Supplementary Table S7.

**Table 4:** Cumulative effect of thrombophilic variants on the risk of FGR.

Number of Thrombophilic Variants	FGR (*n* = 240) *n* (%)	Control (*n* = 240) *n* (%)	OR (95% CI)	q (BH)	*p*
0 (reference)	25 (10.4)	74 (30.8)	1.00		–
1	76 (31.7)	106 (44.2)	2.12 (1.24–3.64)	0.006	0.006
2	106 (44.2)	56 (23.3)	5.60 (3.21–9.78)	5.63 × 10^−10^	<0.001
3	33 (13.8)	4 (1.7)	24.42 (7.87–75.78)	1.24 × 10^−10^	<0.001
**Specific combinations**					
PAI-1 4G + *MTHFR* 677T	98 (40.8)	39 (16.2)	3.56 (2.32–5.46)	5.27 × 10^−9^	<0.001
PAI-1 4G + Factor V Leiden	65 (27.1)	13 (5.4)	6.49 (3.46–12.14)	2.27 × 10^−10^	<0.001
*MTHFR* 677T + Factor V Leiden	42 (17.5)	16 (6.7)	2.97 (1.62–5.45)	4.53 × 10^−4^	<0.001
All three variants ^a^	33 (13.8)	4 (1.7)	9.41 (3.28–27.00)	6.03 × 10^−7^	<0.001

Overall *p* value for the distribution of the number of variants: χ^2^ = 67.36; df = 3; *p* < 0.001. Confidence intervals were calculated by the Baptista–Pike method. ^a^ For the rows in the “Specific combinations” section, the reference group comprises all other women (carriers of other combinations or of no variant), unlike the upper section of the table, in which the reference is the absence of any variant; two different odds ratios are therefore reported for the coexistence of all three variants (24.42 and 9.41, respectively). Mean birth weight centile by number of variants: 0 variants, 41.4; 1 variant, 31.3; 2 variants, 21.0; 3 variants, 10.6 (Spearman ρ = −0.406; *p* < 0.001). q, Benjamini–Hochberg false discovery rate applied within this family of tests; Bonferroni-adjusted *p* values for all 36 comparisons are given in Supplementary Table S7.

**Table 5:** Multivariable logistic regression for the prediction of FGR.

Variable	Adjusted OR (95% CI)	*p*
Factor V Leiden carrier	3.33 (2.03–5.47)	<0.001
PAI-1 4G carrier	2.83 (1.89–4.24)	<0.001
*MTHFR* 677T carrier	2.00 (1.35–2.97)	<0.001
Pre-gestational BMI (per 1 kg/m^2^)	1.15 (1.06–1.25)	0.001
Prior hypertensive disease of pregnancy	2.21 (0.74–6.58)	0.155
Smoking	1.48 (0.56–3.89)	0.426
Pre-gestational diabetes	1.05 (0.43–2.53)	0.917
Maternal age (per 1 year)	1.01 (0.96–1.06)	0.701
Multiparity	0.95 (0.59–1.52)	0.836
Chronic hypertension	0.35 (0.08–1.54)	0.164

Area under the ROC curve = 0.736; McFadden pseudo-R^2^ = 0.131; Nagelkerke pseudo-R^2^ = 0.221; *n* = 480. Reduced model (three polymorphisms + BMI): area under the ROC curve = 0.728. BMI, body mass index; OR, odds ratio.

**Table 6:** Multiple linear regression—dependent variable: birth-weight centile.

Variable	β, Birth-Weight Centile (95% CI)	*p*
PAI-1 4G carrier	−12.34 (−17.20 to −7.49)	<0.001
*F5* A carrier	−10.23 (−15.96 to −4.50)	<0.001
*MTHFR* 677T carrier	−8.05 (−12.80 to −3.29)	<0.001

**Table 7:** Perinatal outcomes.

Parameter	FGR (*n* = 240)	Control (*n* = 240)	*p*
Gestational age at delivery, weeks	34.05 ± 3.78	39.02 ± 1.63	<0.001
Preterm birth < 37 weeks, *n* (%)	169 (70.4)	7 (2.9)	<0.001
Delivery < 32 weeks, *n* (%)	66 (27.5)	0 (0.0)	<0.001
Birth weight, g	1819.21 ± 557.63	3386.19 ± 287.88	<0.001
Birth weight centile	6.13 ± 2.28	50.50 ± 23.75	<0.001
Caesarean section, *n* (%)	139 (57.9)	90 (37.5)	<0.001
5 min Apgar score	7.98 ± 1.80	9.30 ± 0.86	<0.001
Apgar score < 7, *n* (%)	52 (21.7)	7 (2.9)	<0.001
Neonatal respiratory support, *n* (%)	73 (30.4)	6 (2.5)	<0.001
NICU admission > 24 h, *n* (%)	93 (38.8)	11 (4.6)	<0.001

Because gestational age at delivery differs by design between the two groups, these comparisons describe the clinical burden of the FGR phenotype rather than an independent effect of growth restriction. FGR, fetal growth restriction; NICU, neonatal intensive care uni.

**Table 8:** Perinatal outcomes by severity of growth restriction.

Parameter	Control (*n* = 240)	5th–10th Centile (*n* = 151)	3rd–5th Centile (*n* = 77)	<3rd Centile (*n* = 12)	*p*
Birth weight centile	50.5 ± 23.8	7.6 ± 1.4	4.0 ± 0.6	1.5 ± 0.8	<0.001
Birth weight, g	3386.2 ± 287.9	1822.3 ± 537.3	1884.5 ± 578.7	1361.7 ± 498.1	<0.001
Gestational age at delivery, weeks	39.0 ± 1.6	33.8 ± 3.6	34.8 ± 4.0	32.6 ± 3.8	<0.001
5 min Apgar score	9.3 ± 0.9	8.0 ± 1.8	8.1 ± 1.8	7.2 ± 2.0	<0.001
Caesarean section, *n* (%)	90 (37.5)	92 (60.9)	38 (49.4)	9 (75.0)	<0.001
Respiratory support, *n* (%)	6 (2.5)	49 (32.5)	19 (24.7)	5 (41.7)	<0.001
NICU admission > 24 h, *n* (%)	11 (4.6)	60 (39.7)	27 (35.1)	6 (50.0)	<0.001

Continuous variables were compared using the Kruskal–Wallis test and categorical variables using the χ^2^ test. NICU, neonatal intensive care unit.

## Data Availability

The de-identified analysis dataset and the command log from which every table and figure in this article was produced are available from the corresponding author on reasonable request, and will be deposited in a public repository with a DOI upon acceptance. Supplementary Table S11 lists every value reported in more than one place in this article, together with the result of a cross-check of each against the analysis dataset; Supplementary Table S6 states when each analysis was performed.

## Supplementary Materials

The following supporting information can be downloaded at https://www.mdpi.com/article/10.3390/biomedicines14102238/s1. Table S1: participant selection and ascertainment; Table S2: Sensitivity analysis–phenotype restricted to below the 5th centile; Table S3: Hardy–Weinberg equilibrium, both groups and all three polymorphisms; Table S4: diagnostics of the multivariable logistic regression model; Table S5: statistical power; Table S6: provenance and timing of the analyses; Table S7: multiplicity-adjusted *p* values for all 36 comparisons; Table S8: birth-weight centile by number of thrombophilic variants; Table S9: Doppler-characterised series from the same department; Table S10: quantitative bias analysis (E-values); Table S11: cross-check of every value reported in more than one place; Figure S1: calibration plot of the multivariable model; Figure S2: Doppler phenotype and effect size by case definition.

## Abbreviations

The following abbreviations are used in this manuscript:

ALIFE	Antithrombotic therapy in patients with recurrent pregnancy loss and inherited thrombophilia
aOR	Adjusted Odds Ratio
ANOVA	Analysis of Variance
CI	Confidence Interval
DNA	Deoxyribonucleic Acid
FIGO	International Federation of Gynecology and Obstetrics
FGR	Fetal Growth Restriction
FVL	Factor V Leiden
FRUIT	Low-molecular-weight heparin for prevention of recurrent pregnancy loss in women with thrombophilia
GA	Gestational Age
MCA	Middle Cerebral Artery
*MTHFR*	Methylenetetrahydrofolate Reductase
NICHD	National Institute of Child Health and Human Development
OR	Odds Ratio
PAI-1	Plasminogen Activator Inhibitor Type 1
PCR	Polymerase Chain Reaction
PlGF	Placental Growth Factor
ROC	Receiver Operating Characteristic
*SERPINE1*	Serpin Family E Member 1 gene
sFlt-1	Soluble fms-like Tyrosine Kinase-1
TIPPS	Thrombophilia in Pregnancy Prophylaxis Study
TORCH	Toxoplasmosis, Other infections, Rubella, Cytomegalovirus, Herpes simplex
