# Supplementary material for: PAI-1, MTHFR, and FVL Thrombophilic Polymorphisms as Risk Factors for Fetal Growth Restriction: A Case–Control Study from a Tertiary Care Center
Source: Biomedicines. 2026 Oct 3;14(10):2238. doi: 10.3390/biomedicines14102238 (PMC13701273; doi:10.3390/biomedicines14102238)
Supplement: Supplementary file 1 [file biomedicines-14-02238-s001.zip › biomedicines-4522925-supplementary.pdf]

Supplementary Materials

PAI-1, MTHFR, and FVL Thrombophilic Polymorphisms as Risk Factors for Fetal Growth Restriction: A Case–Control Study from a Tertiary Care Center

Supplementary tables are numbered in order of first citation in the main text. Gene symbols are italicised (*SERPINE1*, *MTHFR*, *F5*); protein names and the conventional designations of the variants (PAI-1 4G/5G, factor V Leiden) are not. The rs1799889 insertion/deletion polymorphism in the promoter of *SERPINE1* is designated PAI-1 4G/5G throughout, as in the main text, tables and figures.

Table S1. Participant selection and ascertainment

Genotyping was performed during clinical care, on individual clinical referral, in external private laboratories accredited by the national health authority and recognised by the hospital in which the pregnancies were monitored. Because these investigations were ordered and reported outside the hospital laboratory information system, the total number of pregnancies delivered in the study period that were, and were not, genotyped could not be reconstructed retrospectively, and a flow diagram with source-population denominators cannot be presented. The sampling frame is described instead.

Element	Description
Source population	Singleton pregnancies delivered in the Department of Obstetrics and Gynaecology, Craiova County Emergency Clinical Hospital, 2021–2025
Ascertainment	Pregnancies for which genotyping of all three polymorphisms had been performed during clinical care and whose reports were retrievable from the hospital record
Cases	Birth-weight centile below the 10th, complete genotyping and complete perinatal data, after the exclusion criteria of Section 2.2 (n = 240; 151 at the 5th–10th, 77 at the 3rd–5th and 12 below the 3rd centile)
Controls	Birth-weight centile between the 10th and the 90th, complete genotyping, delivered in the same period (n = 240)
Indication for genotyping	Requested individually on clinical grounds by the treating clinician, before the present study was conceived; the indication is recorded in the referral rather than in a research database and could not be classified uniformly across participants
Denominators	Not reconstructible (see above); reported as a limitation in Section 4.7
Laboratories	Five external private laboratories accredited by the Romanian Ministry of Health; genotype reports only, without run-level data
Blinding	The laboratories were unaware of the study and of case–control status by construction, since testing preceded the study
Record verification	All genotype and perinatal records independently re-checked against the original charts by two investigators; discrepancies resolved against source laboratory reports

Consequence for interpretation: the clinical indication for ordering a thrombophilia panel is unlikely to have been identical in a pregnancy complicated by growth restriction and in one with normal growth. If testing among controls was preferentially requested for women with a personal or family history suggestive of thrombophilia, the control group is enriched for carriers and the odds ratios reported here are biased towards the null. The comparisons across severity strata, which are made within the case group, are not affected by differential testing between cases and controls.

Table S2. Sensitivity analysis — phenotype restricted to below the 5th centile

Provenance. This analysis was not specified before the study. It was performed in response to the first round of review, which asked whether the 5th–10th centile stratum contains a substantial proportion of constitutionally small fetuses. It is therefore a post hoc sensitivity analysis and is reported as exploratory (see Table S6). Cases below the 5th centile (n = 89) were compared with the same 240 controls.

S2a. Unadjusted associations

Variant	All cases (n = 240)	5th–10th centile only (n = 151)	Below the 5th centile (n = 89)
PAI-1 4G carrier	3.02 (2.07–4.40)	2.37 (1.55–3.61)	4.90 (2.75–8.73)
MTHFR 677T carrier	2.07 (1.44–2.97)	1.90 (1.26–2.86)	2.40 (1.46–3.94)
F5 A carrier	3.64 (2.27–5.83)	2.11 (1.23–3.64)	7.78 (4.41–13.75)

S2b. Multivariable model restricted to cases below the 5th centile

Variable	Adjusted OR (95% CI)	p
F5 A carrier	8.37 (4.31–16.25)	3.47×10^{-10}
PAI-1 4G carrier	5.69 (2.86–11.32)	7.05×10^{-7}
MTHFR 677T carrier	3.24 (1.74–6.05)	2.19×10^{-4}
Pre-gestational BMI (per 1 kg/m²)	0.81 (0.68–0.96)	0.017

Adjusted for the same covariates as the primary model (maternal age, pre-gestational body mass index, smoking, parity, chronic hypertension, pre-gestational diabetes and previous hypertensive disease of pregnancy); area under the ROC curve 0.849. Restricting the phenotype strengthens every genotype association, which is the pattern expected when a genuine effect is diluted by non-differential inclusion of constitutionally small fetuses in the mildest stratum.

Note on body mass index. The direction of the association with pre-gestational body mass index is reversed in this restricted model (adjusted OR 0.81; 95% CI 0.68–0.96) relative to the primary model of the whole cohort (adjusted OR 1.15; 95% CI 1.06–1.25). Both estimates are nominally significant. We draw no inference from this reversal: the restricted analysis contains 89 cases, the two models are fitted to different case sets, and maternal adiposity is associated with higher absolute birth weight, so restricting the phenotype to the most severely growth-restricted pregnancies is expected to deplete the case group of women with higher body mass index. This is stated in Section 4.7 of the main text.

Table S3. Hardy–Weinberg equilibrium, both groups and all three polymorphisms

Polymorphism	Group	Observed (wt/het/var)	Expected	χ^2	p (χ^2)	p (exact)	F
PAI-1 4G/5G	Cases	70 / 106 / 64	63.0 / 119.9 / 57.0	3.24	0.072	0.0716	0.116
PAI-1 4G/5G	Controls	133 / 81 / 26	125.4 / 96.1 / 18.4	5.96	0.0147	0.0158	0.158
MTHFR C677T	Cases	103 / 116 / 21	108.0 / 106.0 / 26.0	2.14	0.1435	0.1877	−0.094
MTHFR C677T	Controls	146 / 78 / 16	142.6 / 84.8 / 12.6	1.54	0.2147	0.2047	0.080
F5 G1691A	Cases	160 / 79 / 1	165.8 / 67.3 / 6.8	7.21	0.0073	0.0049	−0.173
F5 G1691A	Controls	211 / 29 / 0	211.9 / 27.2 / 0.9	0.99	0.3192	1.000	−0.064

wt, homozygous wild type; het, heterozygous; var, homozygous variant; F, fixation index (positive values indicate a deficit of heterozygotes). Exact p values were computed by the method of Wigginton et al. In the control group, distributions conformed to Hardy–Weinberg equilibrium for *MTHFR* C677T and *F5* G1691A, with a significant departure only for PAI-1 4G/5G, in the form of a heterozygote deficit (81 observed vs. 96.1 expected; F = 0.158). In the case group, the departure at *F5* G1691A (exact p = 0.005) takes the form of an excess of heterozygotes together with a deficit of homozygotes (1 observed vs. 6.8 expected); the heterozygote excess is expected under the alternative hypothesis of a genuine association, since cases are sampled conditionally on genotype, whereas the shortfall of homozygotes rests on a single observation and is uninformative. The six exact p values in this table are those reported in the footnote of Table 2 and in Sections 3.2 and 4.7 of the main text.

Table S4. Diagnostics of the multivariable logistic regression model

Diagnostic	Result	Interpretation
Events per variable	240 events / 10 covariates = 24	well above the conventional minimum of 10
Covariate selection	chosen on clinical grounds; no stepwise or data-driven selection	the covariate set was not documented before the analysis (Table S6)
Missing data	1 value in 480 participants (chronic hypertension)	coded as absent; no imputation required
Multicollinearity (VIF)	all ≤ 1.19	no material collinearity
Linearity in the logit (Box–Tidwell)	maternal age p = 0.160; BMI p = 0.921	linearity assumption satisfied
Calibration (Hosmer–Lemeshow)	χ^2 = 9.69, df = 8, p = 0.288	no evidence of miscalibration
Brier score	0.2081	—
Influential observations (Cook's D)	22 observations exceed 4/n = 0.0083	exclusion strengthens the estimates (aOR 3.89, 3.61, 2.39)
Apparent area under the ROC curve	0.736	as reported in Table 5 and Figure 8
Bootstrap-corrected AUC (1000 resamples)	0.713 (optimism 0.0225)	internally validated; reported in Section 3.5
Model without the three genotypes	AUC 0.614	—
Likelihood ratio test for the genotype block	χ^2 = 72.2, df = 3, p = 1.44×10^{-15}	genotype contributes independently of maternal covariates

S4a. Variance inflation factors

Covariate	VIF	Covariate	VIF
F5 A carrier	1.05	Pre-gestational diabetes	1.02
PAI-1 4G carrier	1.03	Maternal age	1.03
MTHFR 677T carrier	1.01	Multiparity	1.18
Pre-gestational BMI	1.09	Chronic hypertension	1.11
Previous hypertensive disease of pregnancy	1.02	Smoking	1.19

S4b. Interaction between the polymorphisms (departure from additivity on the log-odds scale)

Interaction term	Interaction OR	p
PAI-1 4G × *MTHFR* 677T	1.86	0.132
PAI-1 4G × *F5* A	3.28	0.026
MTHFR 677T × *F5* A	0.48	0.145
All three terms jointly (likelihood ratio, 3 df)	—	0.045

A positive interaction was observed between the PAI-1 4G and factor V Leiden alleles (interaction OR 3.28; p = 0.026), and the three interaction terms were jointly significant at the conventional threshold (p = 0.045). Neither result survives adjustment for the three interaction tests performed (Bonferroni-adjusted p = 0.078). The data are therefore compatible with, but do not establish, a supra-additive effect of coexisting PAI-1 4G and factor V Leiden alleles, and we describe the finding as a graded association with variant load rather than as demonstrated biological synergism. These interaction tests were performed in response to the first round of review and are exploratory (Table S6); they are reported in Section 3.5 of the main text.

Table S5. Statistical power

Provenance. The study was assembled retrospectively from pregnancies already genotyped during clinical care; the sample size was determined by the number of eligible records and was not fixed by a power calculation. The calculations below were

performed after the analyses, in response to the first round of review, and are presented to document the resolution of the study rather than its design (Table S6). The first row of the table below is a design-stage calculation reported for reference only: to detect an odds ratio of 2.0 under the dominant model at a two-sided α of 0.05 with 80% power, 133 participants per group would be required for the PAI-1 4G allele (expected carrier prevalence 45%), 133 for the *MTHFR* 677T allele (40%) and 245 for factor V Leiden (12%). The achieved sample of 240 per group therefore exceeds that reference requirement for two of the three variants and falls 5 participants short for factor V Leiden, whose observed effect is nevertheless far larger than the reference value of 2.0.

Comparison	Carrier prevalence in controls	Observed OR	Power (α = 0.05)	Power (α = 0.05/14)	Minimum detectable OR, 80% power	Minimum detectable OR, 90% power
PAI-1 4G carrier	44.6%	3.02	> 99.9%	99.9%	1.67	1.81
MTHFR 677T carrier	39.2%	2.07	97.8%	85.1%	1.67	1.81
F5 A carrier	12.1%	3.64	> 99.9%	99.7%	2.01	2.21
PAI-1 4G/4G (recessive)	10.8%	2.99	99.4%	94.0%	2.06	2.28
MTHFR 677TT (recessive)	6.7%	1.34	13.6%	2.0%	2.38	2.67
MTHFR TT vs. CC	9.9%	1.86	42.4%	13.1%	2.57	2.92

Power was computed from the carrier frequencies actually observed in the control group. The α = 0.05/14 column applies the Bonferroni threshold for the 14 tests of Table 2. The study resolves the dominant model of all three polymorphisms at both thresholds; it is clearly underpowered for the recessive model of *MTHFR* C677T, which is the explanation for the non-significant result at that locus and is stated as such in Sections 3.2 and 4.3 of the main text. The values in the first two columns of power are those quoted in Section 2.6.

Table S6. Provenance and timing of the analyses

No analysis plan was registered before the study, and the study was assembled retrospectively from clinical records. The table below states, for every analysis reported, when it was first performed and whether it was planned before the data were examined. It is provided so that no analysis in this manuscript is read as pre-specified unless it is described as such here.

Analysis	First reported	Status
Comparison of genotype frequencies, dominant model	Original submission	Primary analysis; chosen on clinical grounds before the data were analysed, but not documented in advance
Codominant, recessive and allelic models	Original submission	Secondary, exploratory
Stratification by severity of restriction	Original submission	Planned before analysis; not documented in advance
Cumulative variant-load analysis	Original submission	Planned before analysis; not documented in advance
Multivariable logistic regression	Original submission	Covariates chosen on clinical grounds, no data-driven selection; the covariate set was not documented in advance
Multiple linear regression on birth-weight centile	Original submission (table footnote); promoted to the primary weight analysis in revision 1	Substituted for the gestational-age-adjusted weight model on causal grounds during revision
Hardy–Weinberg exact tests	Revision 1, at the request of Reviewer 1	Post hoc
Benjamini–Hochberg and Bonferroni corrections	Revision 1, at the request of Reviewers 1 and 2	Post hoc
Power calculations	Revision 1, at the request of Reviewer 2	Post hoc
Model diagnostics and bootstrap internal validation	Revision 1, at the request of Reviewer 3	Post hoc

Analysis	First reported	Status
Pairwise interaction terms	Revision 1, at the request of Reviewer 3	Post hoc, exploratory
Restriction of the case definition to below the 5th centile	Revision 1, at the request of Reviewer 2	Post hoc sensitivity analysis
Within-case correlation and post-hoc comparisons	Revision 1, at the request of Reviewer 3	Post hoc
E-values for unmeasured confounding	Revision 1, at the request of Reviewer 2	Post hoc
Doppler-characterised series of 80 pregnancies	Revision 1, at the request of Reviewer 2	Post hoc; external to the main cohort
Cross-check of all repeated values (Table S11)	Revision 2	Documentation, not an analysis

Table S7. Multiplicity-adjusted p values for all 36 comparisons

Benjamini–Hochberg false discovery rate (q) was applied within each family of tests and, as a sensitivity analysis, across all 36 comparisons reported in Tables 2, 3 and 4. Bonferroni-adjusted p values are given for the same families. These corrections were applied in response to the first round of review (Table S6).

S7a. Table 2 — genotype and allele models (14 tests)

Comparison	Cases	Controls	OR (95% CI)	p	q (BH)	p (Bonferroni)
PAI-1 4G/5G vs 5G/5G	106/176	81/214	2.49 (1.65–3.74)	1.17×10^{-5}	2.10×10^{-5}	1.64×10^{-4}
PAI-1 4G/4G vs 5G/5G	64/134	26/159	4.68 (2.73–8.03)	6.85×10^{-9}	3.83×10^{-8}	9.60×10^{-8}
PAI-1 4G carrier (dominant)	170/240	107/240	3.02 (2.07–4.40)	8.21×10^{-9}	3.83×10^{-8}	1.15×10^{-7}
PAI-1 4G/4G (recessive)	64/240	26/240	2.99 (1.82–4.92)	1.20×10^{-5}	2.10×10^{-5}	1.68×10^{-4}
PAI-1 4G allele	234/480	133/480	2.48 (1.90–3.25)	2.50×10^{-11}	3.51×10^{-10}	3.51×10^{-10}
MTHFR CT vs CC	116/219	78/224	2.11 (1.44–3.09)	1.28×10^{-4}	1.80×10^{-4}	0.002
MTHFR TT vs CC	21/124	16/162	1.86 (0.93–3.74)	0.109	0.127	1.000
MTHFR 677T carrier (dominant)	137/240	94/240	2.07 (1.44–2.97)	1.20×10^{-4}	1.80×10^{-4}	0.002
MTHFR TT (recessive)	21/240	16/240	1.34 (0.68–2.64)	0.494	0.494	1.000
MTHFR 677T allele	158/480	110/480	1.65 (1.24–2.20)	7.05×10^{-4}	8.97×10^{-4}	0.010
F5 GA vs GG	79/239	29/240	3.59 (2.24–5.76)	3.26×10^{-8}	9.13×10^{-8}	4.57×10^{-7}
F5 AA vs GG	1/161	0/211	3.95 (0.16–97.69)	0.433	0.466	1.000
F5 A carrier (dominant)	80/240	29/240	3.64 (2.27–5.83)	3.26×10^{-8}	9.13×10^{-8}	4.57×10^{-7}
F5 A allele	81/480	29/480	3.16 (2.02–4.93)	1.49×10^{-7}	3.48×10^{-7}	2.09×10^{-6}

S7b. Table 3 — severity strata (15 comparisons vs. controls)

Comparison vs. controls	OR (95% CI)	p	q (BH)
PAI-1 4G carrier — 5th–10th centile	2.37 (1.55–3.61)	7.24×10^{-5}	1.81×10^{-4}
PAI-1 4G carrier — 3rd–5th centile	4.07 (2.27–7.32)	8.71×10^{-7}	6.53×10^{-6}
PAI-1 4G carrier — < 3rd centile	not estimable [a]	9.04×10^{-5}	1.94×10^{-4}
PAI-1 4G/4G — 5th–10th centile	2.48 (1.42–4.33)	0.002	0.003
PAI-1 4G/4G — 3rd–5th centile	4.20 (2.25–7.83)	1.08×10^{-5}	4.05×10^{-5}
PAI-1 4G/4G — < 3rd centile	2.74 (0.70–10.78)	0.148	0.170
MTHFR 677T carrier — 5th–10th centile	1.90 (1.26–2.86)	0.003	0.004
MTHFR 677T carrier — 3rd–5th centile	3.05 (1.78–5.22)	4.07×10^{-5}	1.22×10^{-4}
MTHFR 677T carrier — < 3rd centile	0.52 (0.14–1.96)	0.380	0.407
MTHFR 677TT — 5th–10th centile	2.02 (1.00–4.05)	0.067	0.092
MTHFR 677TT — 3rd–5th centile [b]	0.18 (0.02–1.41)	0.083	0.103
MTHFR 677TT — < 3rd centile	1.27 (0.15–10.49)	0.576	0.576
F5 A carrier — 5th–10th centile	2.11 (1.23–3.64)	0.007	0.011
F5 A carrier — 3rd–5th centile	6.73 (3.72–12.17)	2.10×10^{-10}	3.16×10^{-9}
F5 A carrier — < 3rd centile	21.83 (5.58–85.31)	2.61×10^{-6}	1.30×10^{-5}

[a] All 12 pregnancies below the 3rd centile carried the PAI-1 4G allele, so the odds ratio is not estimable; this is the entry shown as "n.e." in Table 3 of the main text. The exact p value is computable and is reported. With a Haldane–Anscombe correction of 0.5 the odds ratio would be 31.05 (95% CI 1.82–530.40); that value is given here only to indicate direction and imprecision and is not used in the main text.

[b] Only one of the 77 pregnancies in the 3rd–5th centile stratum carried the *MTHFR* 677TT genotype, against 16 of 240 controls, so the point estimate is inverse in this cell while carriage of the 677T allele in the same stratum is positively associated (OR 3.05). The contrast rests on a single case, does not reach the conventional threshold, and shows no trend across strata (Cochran–Armitage p = 0.634); it is noted in Section 3.3 of the main text and no inference is drawn from it.

S7c. Table 4 — variant load and combinations (7 comparisons)

Comparison	Cases	Controls	OR (95% CI)	p	q (BH)
1 variant vs none	76/240 (31.7%)	106/240 (44.2%)	2.12 (1.24–3.64)	0.006	0.006
2 variants vs none	106/240 (44.2%)	56/240 (23.3%)	5.60 (3.21–9.78)	2.41×10^{-10}	5.63×10^{-10}
3 variants vs none	33/240 (13.8%)	4/240 (1.7%)	24.42 (7.87–75.78)	1.77×10^{-11}	1.24×10^{-10}
PAI-1 4G + *MTHFR* 677T	98/240 (40.8%)	39/240 (16.2%)	3.56 (2.32–5.46)	3.01×10^{-9}	5.27×10^{-9}
PAI-1 4G + *F5* A	65/240 (27.1%)	13/240 (5.4%)	6.49 (3.46–12.14)	6.48×10^{-11}	2.27×10^{-10}
MTHFR 677T + *F5* A	42/240 (17.5%)	16/240 (6.7%)	2.97 (1.62–5.45)	3.88×10^{-4}	4.53×10^{-4}
All three variants	33/240 (13.8%)	4/240 (1.7%)	9.41 (3.28–27.00)	4.31×10^{-7}	6.03×10^{-7}

S7d. Summary across all 36 comparisons

Criterion	Significant / total
Unadjusted p < 0.05	28 / 36
Benjamini–Hochberg q < 0.05	28 / 36
Bonferroni-adjusted p < 0.05	24 / 36

Four comparisons lose significance under the global Bonferroni threshold and retain it under Benjamini–Hochberg: PAI-1 4G/4G in the 5th–10th centile stratum (p = 0.0015; q = 0.0022; Bonferroni p = 0.054); *MTHFR* 677T carriage in the 5th–10th centile stratum (p = 0.0025; q = 0.0035; Bonferroni p = 0.090); factor V Leiden carriage in the 5th–10th centile stratum (p = 0.0073; q = 0.0094; Bonferroni p = 0.264); and one variant versus none (p = 0.0063; q = 0.0084; Bonferroni p = 0.228). The last of these is the first step of the variant-load gradient, and its loss of significance under the most conservative correction is stated in Sections 3.4 and 4.5 of the main text. All three primary associations — PAI-1 4G, *MTHFR* 677T and factor V Leiden carriage — remain significant under both correction methods.

Table S8. Birth-weight centile by number of thrombophilic variants (Figure 6)

Number of variants	n	Median centile (IQR)	Mean centile
0	99	39.7 (10.2–69.0)	41.4
1	182	18.9 (7.7–54.1)	31.3
2	162	7.8 (4.4–28.1)	21.0
3	37	6.0 (3.9–7.2)	10.6

Kruskal–Wallis across the four groups: $H = 79.90$, $df = 3$, $p = 3.23 \times 10^{-17}$. Dunn's post-hoc comparisons with Holm adjustment:

Comparison	z	p	p (Holm)	Marker in Figure 6
0 vs 1 variants	3.07	0.002	0.004	**
0 vs 2 variants	7.05	1.83×10^{-12}	9.16×10^{-12}	**
0 vs 3 variants	7.14	9.50×10^{-13}	5.70×10^{-12}	**
1 vs 2 variants	4.77	1.84×10^{-6}	5.52×10^{-6}	**
1 vs 3 variants	5.50	3.82×10^{-8}	1.53×10^{-7}	**
2 vs 3 variants	2.61	0.009	0.009	**

Markers: **p < 0.01;** ** p < 0.0001. All six pairwise comparisons are significant after Holm adjustment; the comparison between two and three coexisting variants, which was previously displayed as non-significant, is significant at p = 0.009. Figure 6 has been redrawn so that its markers correspond to the values in this table.

Within-case analysis (structural component removed)

Because Figure 6 pools cases and controls, and case status was itself defined by centile, part of the association is imposed by the sampling scheme. The analysis was therefore repeated within the case group alone, where this circularity does not arise. Among the 240 growth-restricted pregnancies, the correlation between the number of variants and birth-weight centile was Spearman $\rho = -0.315$ ($p < 0.001$), with mean centile falling from 7.8 in women carrying no variant to 6.7, 5.6 and 5.4 in those carrying one, two and three variants (Kruskal–Wallis $H = 26.18$, $p < 0.001$). Within the control group the correlation was absent ($\rho = -0.041$; $p = 0.53$). The graded relationship therefore persists after removal of the case–control structure.

Table S9. Doppler-characterised series from the same department

Eighty pregnancies from the same department, ascertained with the same severity strata and genotyped for the same three polymorphisms, in whom uterine and umbilical artery Doppler indices and the presence of a protodiastolic notch were recorded. Uteroplacental abnormality was defined as a third-trimester notch or a uterine or umbilical artery pulsatility index above the 95th centile of the controls of that series. This series is external to the main cohort of 480 and was assembled for a different study; it is presented as supporting context for the phenotype, not as part of the primary analysis.

Index	Controls (n = 40)	5th–10th centile (n = 25)	3rd–5th centile (n = 13)	< 3rd centile (n = 2)
Uterine artery PI, third trimester — median	0.84	0.89	1.22	1.25
Umbilical artery PI, third trimester — median	1.04	1.10	1.82	1.82
Third-trimester notch, n (%)	1/40 (2%)	1/25 (4%)	6/13 (46%)	2/2 (100%)
Middle cerebral artery PI < 10th centile, n (%)	10/40 (25%)	7/25 (28%)	10/13 (77%)	2/2 (100%)
Any uteroplacental abnormality, n (%)	3/40 (8%)	2/25 (8%)	9/13 (69%)	2/2 (100%)

S9a. Comparisons with controls

Comparison	Result
5th–10th centile vs. controls — uterine artery PI T3	0.89 vs. 0.84; Mann–Whitney p = 0.63
5th–10th centile vs. controls — umbilical artery PI T3	1.10 vs. 1.04; p = 0.57
5th–10th centile vs. controls — third-trimester notch	1/25 vs. 1/40; Fisher p = 1.00
5th–10th centile vs. controls — any uteroplacental abnormality	2/25 vs. 3/40; Fisher p = 1.00
Below the 5th centile vs. controls — any uteroplacental abnormality	11/15 vs. 3/40; OR 33.9; p < 0.0001
Below the 5th centile vs. controls — third-trimester notch	8/15 vs. 1/40; p < 0.0001
Below the 5th centile vs. controls — MCA PI < 10th centile	12/15 vs. 10/40; OR 12.0; p = 0.0004
Below the 5th centile vs. controls — uterine artery PI T3	1.25 vs. 0.84; p = 0.0003
Below the 5th centile vs. controls — umbilical artery PI T3	1.82 vs. 1.04; p = 0.0002

S9b. Genotype and Doppler within the same series

Variant	Any uteroplacental abnormality	Third-trimester notch	MCA PI < 10th centile
F5 A carrier	OR 3.67; p = 0.041	9/30 vs 1/50; OR 21.0; p = 0.0005	OR 2.57; p = 0.058
PAI-1 4G carrier	14/49 vs 2/31; OR 5.80; p = 0.021	OR 2.83; p = 0.30	OR 3.69; p = 0.017
MTHFR 677T carrier	OR 0.50; p = 0.26	OR 0.44; p = 0.31	OR 1.07; p = 1.00

Note on *MTHFR*. In this series the point estimates for *MTHFR* 677T carriage are below unity for uteroplacental abnormality and for notching, in the opposite direction to the association with growth restriction observed in the main cohort. None reaches the conventional threshold, and the series contains 80 pregnancies, so the estimates are imprecise; but the Doppler data do not support a placental-vascular mechanism for *MTHFR* in the way they do for factor V Leiden and PAI-1. This is stated in Sections 4.3 and 4.7 of the main text and is one reason the *MTHFR* association is interpreted as a marker in a specific population rather than as evidence of a vascular mechanism.

Limitations of this series. It is small; it was assembled for a study of hypertensive disease of pregnancy and is therefore enriched for preeclampsia (23/25 of the 5th–10th centile stratum, predominantly late-onset); the < 3rd centile stratum contains two pregnancies; first-trimester uterine artery values show a narrow, implausible distribution and were not used; and middle cerebral artery PI below the 10th centile was recorded in 25% of controls, indicating a calibration problem in the reference curve, so only between-stratum contrasts are reported for that index.

Table S10. Quantitative bias analysis for an unmeasured confounder

Hypertensive disease of the index pregnancy was not recorded in the research database. The E-value quantifies how strong an unmeasured confounder would have to be, on the risk-ratio scale and conditional on the measured covariates, to explain away each adjusted association.

Variant	Adjusted OR	E-value for the point estimate	Lower confidence limit	E-value for the confidence limit
Factor V Leiden	3.33	6.12	2.03	3.48
PAI-1 4G	2.83	5.11	1.89	3.19
MTHFR 677T	2.00	3.41	1.35	2.04

To explain away the adjusted association with factor V Leiden, an unmeasured confounder would have to be associated with both the genotype and fetal growth restriction by a risk ratio of at least 6.12 each; to move the confidence interval to include the null, at least 3.48 each. Published associations between hypertensive disease of pregnancy and these polymorphisms are of the order of 1.2–1.8, and between hypertensive disease and growth restriction of the order of 2–3. Both conditions must be met simultaneously for a confounder to explain an association, and the exposure-side association is the smaller of the two, so preeclampsia is unlikely on its own to account for the associations reported here. The margin is narrowest for *MTHFR* 677T, where the E-value for the confidence limit is 2.04 and lies within the range reported for the confounder–outcome association; the association with *MTHFR* is accordingly the least robust of the three to unmeasured confounding by hypertensive disease. This analysis does not replace measurement of the variable. Both ranges, and this qualification, are stated in Section 4.7 of the main text.

Table S11. Cross-check of every value reported in more than one place

Every quantity that appears in more than one location in the manuscript, the figures or these supplementary materials was recalculated from the analysis dataset and checked against every location in which it appears. The table below records the result of that check. It is provided in response to the reviewer's observation that the manuscript has shown inconsistencies between components across two rounds of review.

Quantity	Value recalculated from the dataset	Locations checked	Agreement
Cases / controls	240 / 240	Abstract, §2.1, §3.1, Tables 1–8, Table S1	all
Severity strata	151 / 77 / 12	§2.3, §3.1, Table 3, Table 8, Figures 4, 9, 10, Table S1	all
Maternal age	26.40 ± 4.38 / 26.22 ± 4.12	§3.1, Table 1	all
Pre-gestational BMI	23.94 ± 2.65 / 23.26 ± 2.32	§3.1, Table 1	all
Birth-weight centile	6.13 ± 2.28 / 50.50 ± 23.75	§3.6, Tables 7, 8	all
Birth weight	1819.21 ± 557.63 / 3386.19 ± 287.88	§3.6, Tables 7, 8, Figure 10B	all
Gestational age at delivery	34.05 ± 3.78 / 39.02 ± 1.63	§3.6, Tables 7, 8, Figure 10A	all
5-minute Apgar score	7.98 ± 1.80 / 9.30 ± 0.86	§3.6, Tables 7, 8, Figure 10C	all
Preterm birth < 37 weeks	169 / 7	§3.6, Table 7	all
Delivery < 32 weeks	66 / 0	§2.3, §3.6, Table 7	all
PAI-1 genotype counts	64 / 106 / 70 and 26 / 81 / 133	§3.2, Table 2, Figures 2A, 7, Table S3, Table S7a	all
MTHFR genotype counts	116 / 21 and 78 / 16	§3.2, Table 2, Figure 2B, Tables S3, S7a	all
F5 genotype counts	79 / 1 and 29 / 0	§3.2, Table 2, Figure 2C, Tables S3, S7a	all
OR 3.02, PAI-1 4G carrier	3.02 (2.07–4.40)	Abstract, §3.2, §4.2, Table 2, Figure 3A, Tables S2a, S5, S7a	all
OR 2.07, *MTHFR* 677T carrier	2.07 (1.44–2.97)	Abstract, §3.2, Table 2, Figure 3A, Tables S2a, S5, S7a	all
OR 3.64, factor V Leiden carrier	3.64 (2.27–5.83)	Abstract, §3.2, §4.4, Table 2, Figure 3A, Tables S2a, S5, S7a	all
Adjusted ORs 3.33 / 2.83 / 2.00	as reported	Abstract, §3.5, §4.1, Table 5, Figure 3B, Table S10	all
OR 2.12 / 5.60 / 24.42, variant load	as reported	Abstract, §3.4, §4.5, §5, Table 4, Figure 5B, Table S7c	all
AUC 0.736 (apparent)	0.736	§3.5, Table 5, Figure 8, Table S4	all
AUC 0.713 (bootstrap-corrected)	0.713	§3.5, Table S4	all
β −12.34 / −10.23 / −8.05, centile	as reported	Abstract, §3.5, §4.2, Table 6	all
Hardy–Weinberg exact p, six values	as reported	Table 2 footnote, §3.2, §4.7, Table S3	all
ρ = −0.406 (pooled) and −0.315 (cases)	as reported	§3.4, §4.5, Table 4 footnote, Table S8	all
χ^2 = 67.36, df = 3, variant load	as reported	§3.4, Table 4, Figure 5A	all
89 cases below the 5th centile	77 + 12 = 89	§4.7, Table S2	all
80 pregnancies, Doppler series	40 + 25 + 13 + 2 = 80	§4.7, Table S9, Figure S2	all

The recalculation was performed on the analysis dataset from which every table in the manuscript was produced. All 14 odds ratios of Table 2, all 15 of Table 3 and all 7 of Table 4 were recomputed from the underlying counts and reproduce the published

values; the stratum-specific counts of Table 3 sum exactly to the totals of Table 2; and the Benjamini–Hochberg procedure of Table S7a was re-implemented independently, including the enforcement of monotonicity, and reproduces all 14 q values.

Supplementary figures

Figure S1. Calibration of the multivariable logistic regression model

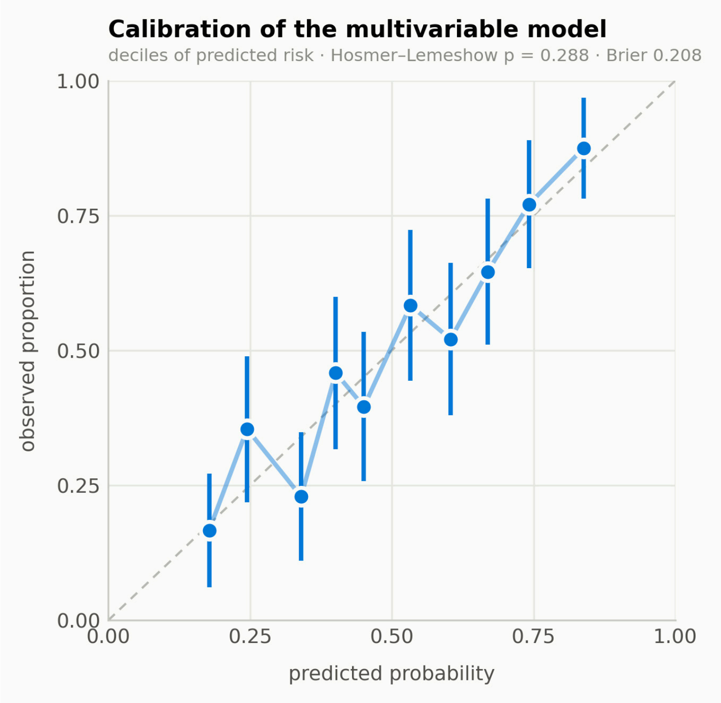

Observed proportion of fetal growth restriction against predicted probability, by decile of predicted risk, with 95% confidence intervals. The dashed line is the line of perfect calibration. Hosmer–Lemeshow $\chi^2 = 9.69$, df = 8, p = 0.288; Brier score 0.208. The diagnostics shown here are those of Table S4.

Figure S2. Doppler phenotype and effect size by definition of the case group

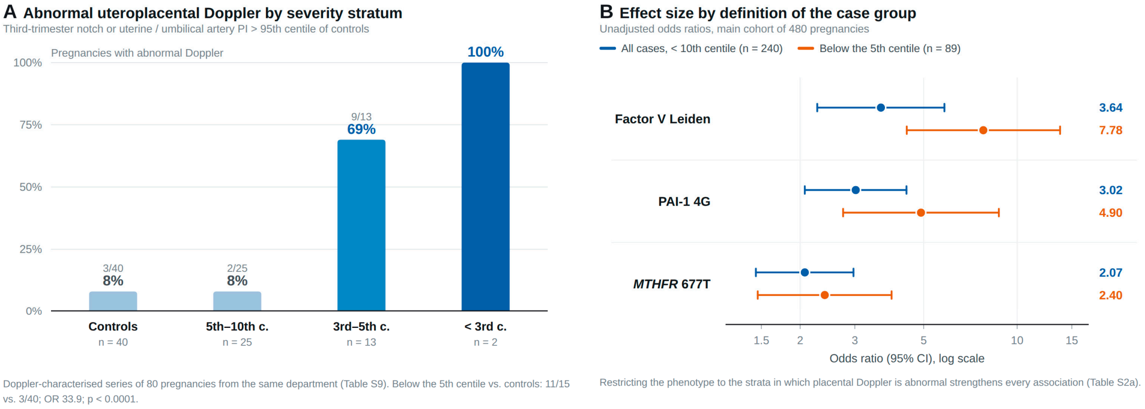

(**A**) Proportion of pregnancies with abnormal uteroplacental Doppler by severity stratum, in the Doppler-characterised series of 80 pregnancies (Table S9). (**B**) Unadjusted odds ratios for the three polymorphisms under the current case definition (all cases below the 10th centile, n = 240) and under the definition restricted to below the 5th centile (n = 89), in the main cohort of 480 (Table S2a). For PAI-1 and factor V Leiden, the strata in which Doppler demonstrates placental disease are the strata in which the genotype association is strongest; for *MTHFR* the Doppler data do not show this pattern (Table S9b). The figure has been redrawn in English for this revision; the underlying values are unchanged.
